# On the fate of pumice rafts formed during the 2012 Havre submarine eruption

**DOI:** 10.1038/ncomms4660

**Published:** 2014-04-22

**Authors:** Martin Jutzeler, Robert Marsh, Rebecca J. Carey, James D. L. White, Peter J. Talling, Leif Karlstrom

**Affiliations:** 1 National Oceanography Centre, Southampton, European Way, Southampton SO14 3ZH, UK; 2 Department of Geology, University of Otago, PO Box 56, Dunedin 9056, New Zealand; 3 Ocean and Earth Science, University of Southampton, National Oceanography Centre, Southampton, European Way, Southampton SO14 3ZH, UK; 4 CODES—ARC Centre of Excellence in Ore Deposits, University of Tasmania, PO Box 79, Hobart 7005, Australia; 5 Department of Geophysics, Stanford University, 397 Panama Mall, Stanford California 94305, USA

## Abstract

Pumice rafts are floating mobile accumulations of low-density pumice clasts generated by silicic volcanic eruptions. Pumice in rafts can drift for years, become waterlogged and sink, or become stranded on shorelines. Here we show that the pumice raft formed by the impressive, deep submarine eruption of the Havre caldera volcano (Southwest Pacific) in July 2012 can be mapped by satellite imagery augmented by sailing crew observations. Far from coastal interference, the eruption produced a single >400 km^2^ raft in 1 day, thus initiating a gigantic, high-precision, natural experiment relevant to both modern and prehistoric oceanic surface dispersal dynamics. Observed raft dispersal can be accurately reproduced by simulating drift and dispersal patterns using currents from an eddy-resolving ocean model hindcast. For future eruptions that produce potentially hazardous pumice rafts, our technique allows real-time forecasts of dispersal routes, in addition to inference of ash/pumice deposit distribution in the deep ocean.

## Abstract (web summary)

Pumice rafts result from volcanic eruptions into and onto water, and can be extensive and potentially hazardous, but tracking their dispersal is difficult. Jutzeler *et al.* combine satellite imagery and an ocean model to accurately forecast pumice raft dispersal routes.

## Graphical abstract

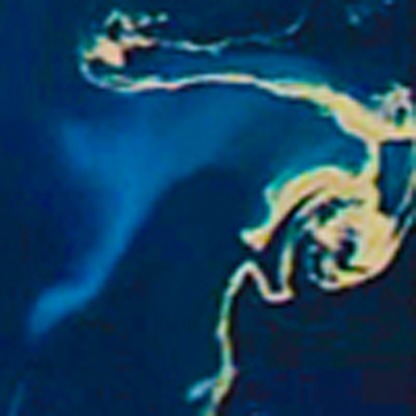

Pumice results from intense vesiculation and quenching of silicic magma. The buoyancy of pumice clasts depends on their size, shape, vesicularity, permeability and temperature when introduced into seawater^1^,^2^,^3^,^4^,^5^,^6^. Centimetre-to-metre thick rafts form when large volumes of buoyant pumice clasts are rapidly released into/onto water. The routes followed by pumice rafts are dependent on source of pumice clasts, initial raft dimensions, oceanic currents and wind direction, nearby coastlines and the ability of pumice clasts to remain buoyant^1^,^3^,^7^,^8^,^9^,^10^,^11^,^12^. Although known to coastal and maritime people since antiquity^7^,^8^,^11^,^12^,^13^,^14^,^15^,^16^,^17^,^18^,^19^,^20^,^21^,^22^, sightings of pumice rafts remain uncommon and spectacular. Before satellite imagery^23^, stranding of pumice clasts on coasts and reports of floating pumice rafts from ships were the only signs of eruptions from uninhabited islands and seamounts^7^,^8^,^13^,^16^,^17^,^24^,^25^,^26^. The sources of pumice rafts previously recognized were shallow (<100 mbsl) seamounts^3^,^11^,^12^,^16^,^26^,^27^ and domes^28^, subaerial pyroclastic flows and fallout^2^,^18^,^19^,^20^,^21^,^22^,^29^, shore erosion and fluvial transport^30^ or volcanic-triggered landslides^25^.

The 2012 eruption of the Havre caldera volcano was the first to unambiguously demonstrate that deep submarine (>700 mbsl)^31^ silicic eruptions can create pumice rafts (Fig. 1). An earthquake swarm of 18 events *M*>3.5 over 12.5 h occurred at the 5-km-wide Havre caldera volcano (31°06′S/179°02′W) on 17 July 2012 coordinated universal time (UTC)^32^. Moderate-Resolution Imaging Spectroradiometer (MODIS) images on 18 and 19 July UTC show an atmospheric plume and a thermal hot spot^31^. The atmospheric plume, probably consisting of steam only, was sourced from a single point, and was not generated by the drifting pumice raft, evidence of the relatively cool temperature of the raft components^33^. In following days, the raft drifted northwest and was separated from its then-inactive source^31^. No raft, plume or discoloured water was produced by the volcano in the next >6 months following the eruption. In mid-August, the raft was up to 60 cm thick and composed of highly vesicular, rounded pumice clasts^31^. In October 2012, a bathymetric survey^31^ revealed a newly constructed 250-m-high cone (>700 mbsl) on the southeast rim of the caldera, possible small aligned cones and a bulge on the 800-m-deep caldera crater wall; youthful pumice was dredged^31^.

The Havre eruption presents us a rare opportunity for testing a large-scale natural particle release experiment in an area where interactions with coastlines and strong boundary currents are negligible. Satellite altimetry reveals a zone of high eddy kinetic energy (EKE)—associated with strong horizontal dispersal—along the eastward-flowing surface currents of the South Tropical Countercurrent^34^, just to the north of the Havre caldera volcano. Observations of surface drift with the local currents are limited to a relatively small number of geo-referenced floats or drifters that have traversed the region, although suitable high-resolution model simulations are now available^35^,^36^,^37^. At basin and global scales, the dispersal implicit in model current data has been evaluated by comparing model ‘particle’ trajectories with selected drifter observations^38^,^39^. In contrast, rare case studies^40^,^41^,^42^ allow for more targeted assessment of models’ regional accuracy and precision.

Here we sample currents from the southwest subtropical Pacific region of an eddy-resolving global ocean model hindcast (NEMO) that spans 1988–2010. NEMO^43^ is a state-of-the-art, portable ocean modelling framework developed by a consortium of European institutions. We use results for the hindcast period to simulate region-typical patterns of particle drift and dispersal (see Methods). Our choice of this hindcast is guided by evidence that eddy-resolving simulations can faithfully reproduce the global EKE field observed with satellite altimetry^44^, whereas lower-resolution eddy-permitting simulations are known to substantially underestimate EKE^38^,^45^. We combine MODIS satellite imagery and hindcast simulation of an eddy-resolving ocean model to track and forecast dispersion of the pumice raft generated by a deep (>700 mbsl) submarine eruption in July 2012 of the Havre volcano in the Kermadec arc (Southwest Pacific). The dispersion of the pumice raft is chiefly dependent on oceanic surface currents and surface winds. This study demonstrates that pumice rafts can be tracked in near-real time, allowing hazard assessment for maritime traffic. Our approach allows prediction of the extent and thickness of pumice clasts and raft-generated tephra deposits on the seafloor, and we also characterize features expected in these deposits.

## Results

### Tracking and observations of the pumice rafts

**Figure 1:**
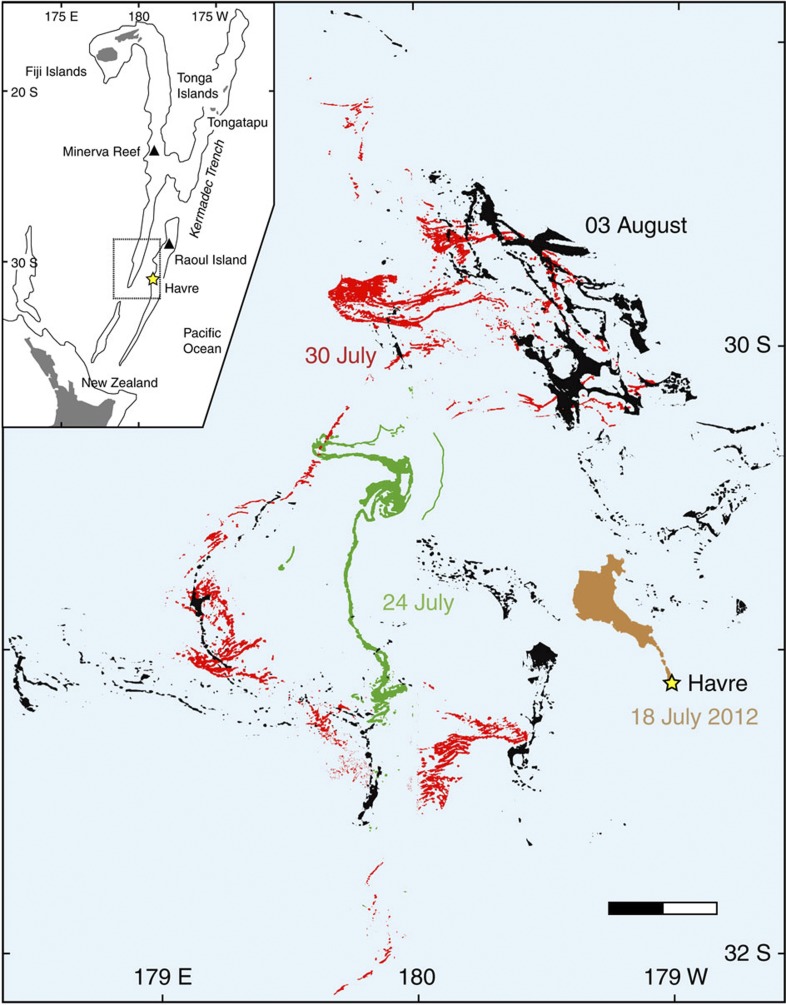
Sequential motion of pumice rafts from MODIS images in the first 3 weeks after the eruption. Images taken from Terra and Aqua satellites at 250 m resolution; raft colours refer to various dates; scale bar, 40 km. Only a few rafts were hidden by cloud cover. Insert shows regional map; islands are in grey; black lines for 2,000 mbsl contour; dashed rectangle shows location of main map.

**Figure 2:**
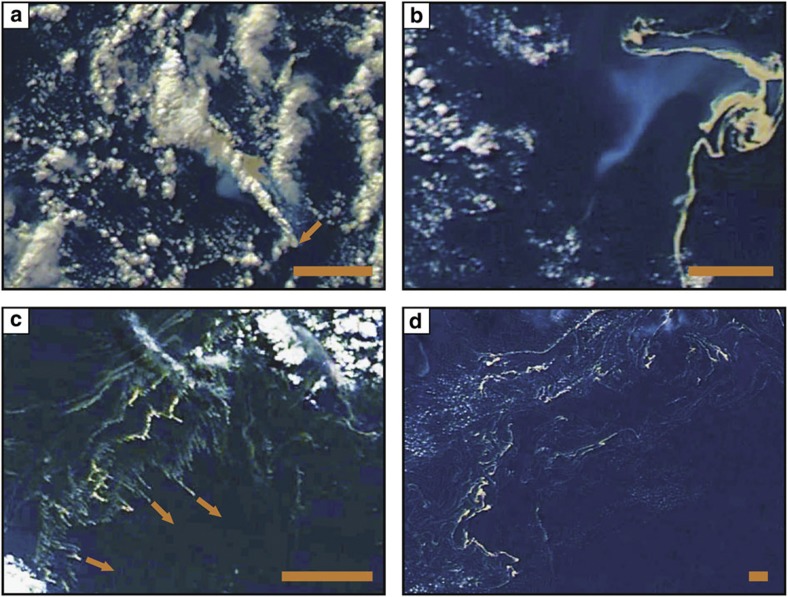
MODIS satellite images of the pumice raft from the July 2012 Havre submarine eruption. (**a**) Syn-eruptive plume (white trail) and pumice raft (yellow) above the submarine vent (arrow) with initial northwest drift (19 July; 01:26 UTC). Note the discoloured water adjacent to the raft (light blue). (**b**) The pumice raft is very elongated and swirls (25 July; 00:50 UTC). Note the persistent discoloured water adjacent to the raft. (**c**) Effect of wind shear and/or oceanic surface currents on elongated bands of pumice rafts. The pumice rafts are dispersed into smaller ribbons of pumice clasts; arrows show a deduced SE-trending wind direction (8 August; 22:08 UTC). (**d**) Pumice raft is widely dispersed and forms very complex dispersal patterns (19 August; 00:44 UTC). Satellite resolution is 250 m; true North is up the page. The original images from MODIS^46^ were filtered (vibrance and saturation) to increase contrasts; scale bars are 20 km.

**Figure 3:**
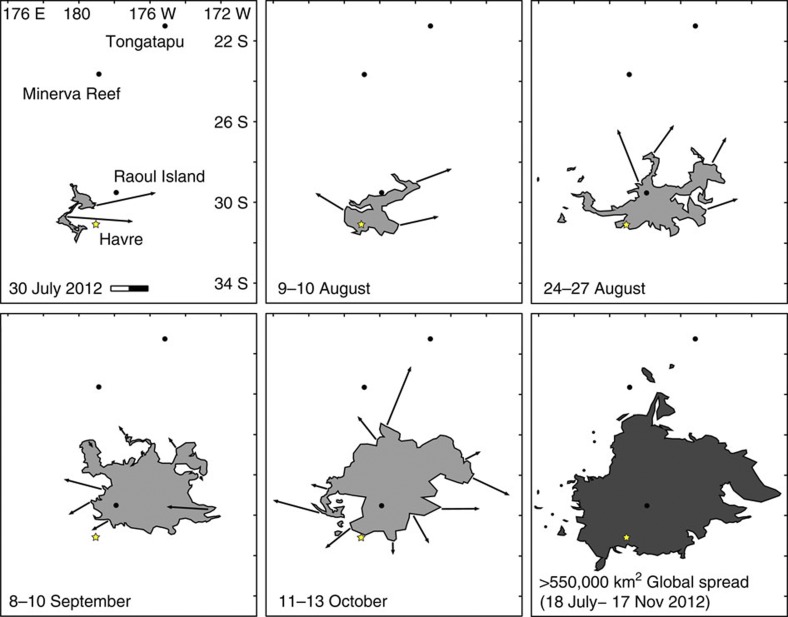
Sequential motion of pumice rafts from MODIS images. Grey polygons show broad areas where pumice clasts are dispersed relative to the Havre volcano. Vectors refer to drift directions to match next raft positions; backward vector on 10 September is due to cloud cover on next date. Main nearby islands of Raoul, Tongatapu and Minerva reef mentioned as landmarks for reference; scale bar 200 km.

**Figure 4:**
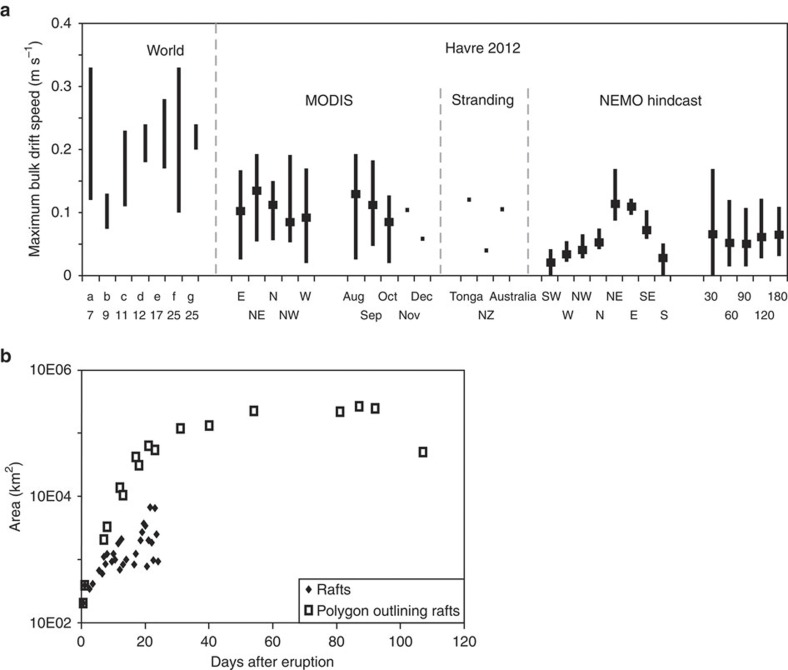
Dispersal rates of rafts from the 2012 Havre eruption compared with NEMO simulations and worldwide data. (**a**) Maximum bulk (straight line) drifting speed of rafts. First arrival or sightings (corresponds to maximum bulk drift speed) for historical rafts^7^,^9^,^11^,^12^,^17^,^25^ (**a**–**g**; numbers refer to reference). The Havre 2012 raft speed is divided by direction of main tongues (east to west) and by months; first strandings; drifting speeds from NEMO simulations for the Havre 2012 raft, per sector and per month. Black lines for speed range, dot for average speed. (**b**) Raft area from analysis of MODIS images. Extent (dispersal) of the raft reaches a plateau after 3–4 weeks of drifting; after 4 months, dilution of the rafts and possible pumice clast sinking does not allow further tracking with MODIS, and dispersal area decreases. Rafts were too complex to identify separately after 11 August.

Using MODIS imagery^46^, we mapped the main raft as it moved and divided over 122 days, from 18 July to 17 November (Fig. 1; Supplementary Figs 1 and 2). Image pixel size (250 m), cloud cover and increasing area of drift with time hampered tracking of the small, complex-shaped rafts (Fig. 2) that prevailed after a few weeks of drifting. Small rafts could be discerned in images acquired up to 22 December 2012 (that is, 157 days after the eruption). The rafts until 3 August could be selected by image analysis and ‘manual’ picking based on RGB colour (Fig. 1), whereas polygons outlining areas containing pumice rafts were manually selected thereafter (Fig. 3; Supplementary Figs 1 and 2). Despite cloud cover impeding raft observations by MODIS imagery, we were able to track the size, number and shape complexity of pumice rafts, which increased with time, until the rafts dissipated by areal diffusion and/or sinking of the clasts. The initial raft was 400 km^2^, and spread to a plateau size of 0.12–0.27 millions km^2^ over a month’s time (Fig. 4), before finally decreasing in size and/or becoming too dilute for MODIS satellite resolution after >3 months. A minimum total pumice raft volume of 0.11–0.16 km^3^ is estimated from colour intensity on MODIS images, assuming a minimum average raft thickness of 50–70 cm just after the eruption, which is an absolute minimum (see Methods). This pumice raft volume is equivalent to 0.03–0.05 km^3^ of dense rock, and more than 3–4 × 10^12^ pumice clasts^12^. However, these numbers are based on few isolated point measurements^31^ during the third week of drifting, and are probably not representative of the thickest rafts at that time of dispersal. In addition, many pumice clasts may have sunk in the first days of drifting, after being partly waterlogged during the eruption^5^ and being abraded thus further reducing their radii and the raft’s packing density. We speculate that the total thickness and volume of the pumice raft was up to five times larger than the minimum values, thus a total pumice raft volume of 1 km^3^ (0.15–0.25 km^3^ dense rock equivalent), an initial raft thickness of up to 3.5 m and >1.5 × 10^13^ clasts^12^. The immensity of the Havre raft, which affected >550,000 km^2^ of ocean (more than twice New Zealand’s subaerial area) in 3 months of drifting, can be compared with the <0.16 km^3^ raft from the 2006 Home Reef eruption^12^ and the 1.25 × 10^−4^ km^3^ of pumice clasts stranded on Australian coasts^11^ 1 year after their eruption in Tonga in 2002. The Havre raft remained mostly coherent during the first week and spread northwest (Fig. 1). Over the next months, it then separated into many domains that spilled in divergent directions (mostly with a northward component), some domains being quickly streamed into >100-km-long tongues (Fig. 3). The fastest bulk drifting speeds (calculated in straight trajectories) were to the northeast and occurred in August (Fig. 4). The Havre raft drifted at 0.02–0.19 m s^−1^ (~2–17 km day^−1^), approaching the bulk drift speeds known globally (calculated from first arrivals) of 0.07–0.28 m s^−1^; global bulk drift speeds (first arrivals) for pumice rafts are 0.20 m s^−1^ on average^7^,^9^,^11^,^12^,^17^,^25^. Estimates of bulk surface current velocities in the NEMO hindcast are in accordance with these speeds, with maximum drift speed at 0.17 m s^−1^ (Fig. 4).

Observations by sailing crews in October 2012 show that pumice rafts get disseminated into narrow, metre-to-kilometre-long single-clast-thick ribbons and isolated pumice clasts. On 5 October, MODIS images show rafts 800 km southwest from Tongatapu island (Fig. 1; Tonga islands, >1,100 km north-northeast from Havre caldera volcano), while ribbons of pumice clasts were encountered as close as 230 km southwest from the island. On 4 November, ribbons of pumice clasts were encountered southwest of Tongatapu, and stranded on local beaches, whereas rafts visible with MODIS were 190 km behind the southwest on the same day. Such major discrepancies between sailor observations and MODIS images indicate complex dispersing effects from both oceanic surface currents and wind shear that extend ribbons of pumice clasts, which become too small or/and thin to be detected with MODIS’ resolution.

Drift and dispersal rates for rafts and ribbons are influenced by their windage (susceptibility to wind) and draft (susceptibility to ocean surface currents). A floating object’s windage and draft will vary with its area and height above water, which for a pumice clast is related to its shape and density (that is, degree of vesicularity and waterlogging). During dispersal, rafts can become elongated and vertically thinned into ribbons. Wind and oceanic surface currents may broaden or degrade rafts by efficiently dispersing pumice clasts into ribbons when blowing/flowing perpendicularly to the thick raft’s length (Fig. 2c). Thin ribbons travelled faster than thick rafts, out-running them by hundreds of kilometres, which implies that wind in this case hindered, rather than helped drive, raft movement.

In early October, sailing crews encountered ribbons of pumice clasts that varied in grain size. Freshly stranded pumice clasts on Tongatapu Island were mostly 5–15 cm in diameter; 200 km southwest of Tongatapu were solitary clasts, or clasts in ribbons of a few m^2^ that consisted of <2 cm clasts. Further (>400 km) southwestward, larger ribbons (a few metres by 200 m long) that comprised coarse pumice clasts (up to 75 cm) were encountered. These observations of contrasting pumice clast sizes suggest clast sorting by grain size, or more precisely by clasts’ individual windage/draft ratio, during transport out of a thick raft.

### Hindcast simulation of eddy-resolving ocean model

**Figure 5:**
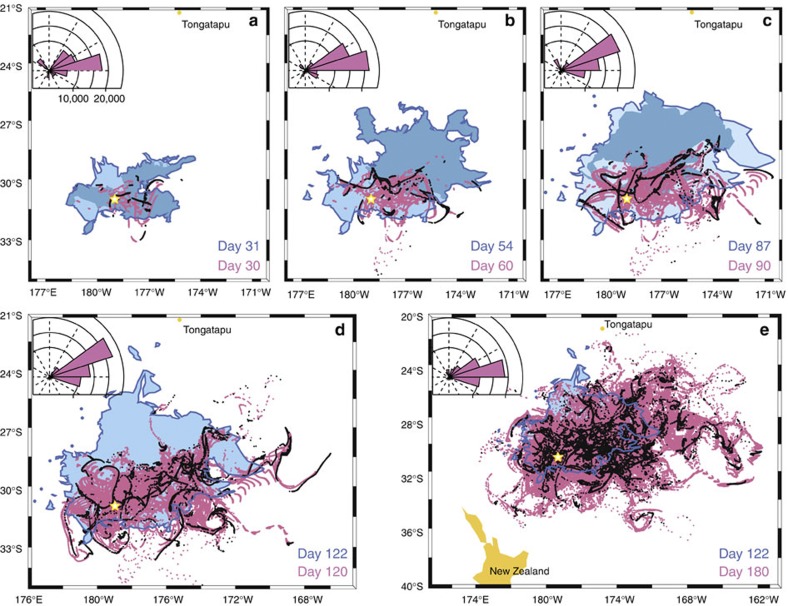
Comparison between MODIS and NEMO particle trajectory data. The comparison shows good fit between observations and simulations. The dispersal of pumice clasts using ARIANE with output from the NEMO 1/12**°** hindcast simulation shows the location of 23 × 2,400 particles after (**a**) 30, (**b**) 60, (**c**) 90, (**d**) 120 and (**e**) 180 days, respectively, in the five panels. Pink dots are past tracks, black dots are last day of simulated drifting. MODIS imagery shows past tracks (light blue polygons with dark blue contour) and last day (medium blue polygons) of data. The Havre caldera volcano (yellow star) and Tongatapu island are shown. For each panel, rose diagrams correspond to the dispersal simulated with NEMO. The radial axis indicates the number of particles (out of 23 × 2,400) per 18**°** sector.

The detailed observations of the 2012 Havre raft provide a rare opportunity to evaluate simulations of oceanic dispersal in an eddying regime, and to predict the dispersal of buoyant objects over the oceans. To isolate the influence on pumice rafts of mean ocean current advection and stirring by oceanic eddies, we determine representative pumice clast trajectories by ‘tracking’ simulated particles with the software package ARIANE ( http://www.stockage.univ-brest.fr/~grima/Ariane/). The simulated particles are entrained in water with motion assigned from output of the NEMO 1/12**°** hindcast (see Methods). On the basis of proposed packing of clasts^12^ in pumice rafts, the 2012 Havre submarine eruption naturally released about 3–15 × 10^12^ pumice clasts (plus ash) in <1 day^31^ from a single point (Supplementary Figs 1 and 2). Evenly distributed in a 717-m^2^ patch centred on the Havre (31°6′50′′S/179°1′10′′W), 100 simulated particles are released at the surface every hour, for 24 h (a total of 2,400 particles) on 18 July of each year in the hindcast (spanning 1988–2010; Supplementary Figs 3–25). The particles are specified as buoyant and are advected in a surface current field that is updated every 5 days. Positions of particles for each year are composited together to account for substantial interannual differences and the chaotic influence of eddies on dispersal. The composite locations of the 2,400 particles for each hindcast year are shown after dispersal over 30, 60, 90, 120 and 180 days (Fig. 5), and compared with MODIS observations during windows of good weather. NEMO is forced with 6-hourly winds supplied by the DFS4.1 (1988–2006) and DFS5.1.1 (2007–2010) data sets^47^. Such frequent updating of the winds is sufficient to resolve individual storms. Year-to-year variations in dispersal over 1988–2010 are thus in part attributed to stochastic (storm related) variability in wind-forced surface drift. Although the pumice raft formed in 2012 and data more recent than 2010 are not yet available, we determined that oceanic conditions in 2012 fell within the 1988–2010 range. The Southwest Pacific region is affected by the El Niño Southern Oscillation, which could contribute to abnormal drifts; however, the El Niño Southern Oscillation index was close to zero for July–December 2012, indicating climatologically neutral winds, supporting that our model of pumice dispersal over July–December 2012 should be ‘contained’ within the 23-year composite of trajectories.

Overall, the hindcast simulation matches most of the pumice drift and directions of propagation described by MODIS imagery, with the exception of northern and northeastern components that affected the raft in the first 3 months (Fig. 5). At 4 months, only a few years in the hindcast model show drift to such low latitudes. By 180 days, dispersal is more indicative of isotropic stirring by eddies, and exceeds most of the MODIS observations at 122 days. Minor strandings of Havre pumice clasts occurred in the Bay of Plenty in April 2013 (North Island, New Zealand, ~260 days), matching the simulated rafts approaching New Zealand at 180 days (Fig. 5). Minor strandings also occurred on the Great Barrier Reef in September and October 2013 (northeast Australia, ~440 days) and were followed by stranding on numerous beaches in New South Wales (east Australia). The ensemble mean advection of the particles (sampling all 23 hindcast years) is encapsulated in rose diagrams (Fig. 5). The vast majority of particles move in an east/northeast direction, again consistent with the MODIS observations. In summary, simulated dispersal is broadly comparable to the MODIS observations, although with discrepancies to the north and northeast owing to some additional influences (such as normal forces and tangential stresses owing to the wind acting on the pumice raft itself, any changes in winds and currents due to vertical momentum fluxes associated with the rafts themselves). To investigate direct forcing of the rafts by local winds, we considered daily mean surface winds in the vicinity of the Havre caldera volcano over 6 months available from the NCEP/NCAR reanalysis project (Supplementary Figs 26–30). The prevailing wind direction is westward for the first 2 months after which synoptic variability increases. There is no clear evidence for Ekman drift in the northward direction (that is, winds blowing northeastwards). Other explanations for the discrepancy between observed and simulated drift include: unresolved submesoscale stirring in the ocean surface layer; rafts acting against winds and currents (see Methods); an unusually strong northeastward component in the oceanic currents of 2012 (not captured in the 1988–2010 hindcast).

### Hazards associated with pumice rafts

**Figure 6:**
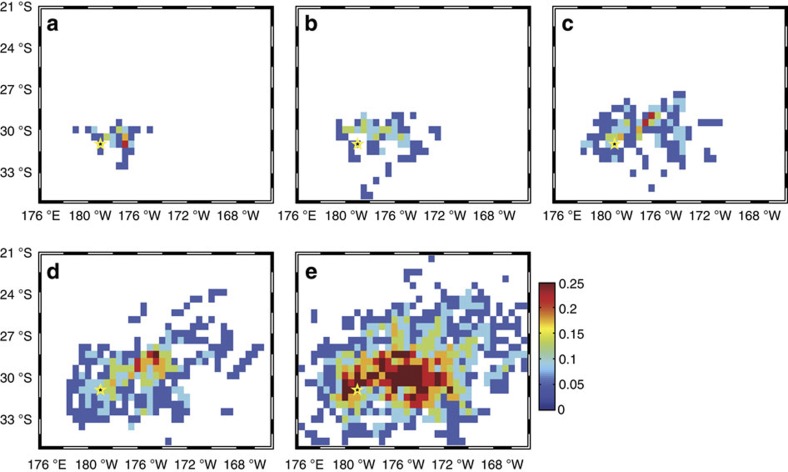
**Likelihood of encountering pumice rafts over time for the 2012 Havre eruption**. Boxes are (**a**) 30, (**b**) 60, (**c**) 90, (**d**) 120 and (**e**) 180 days, and defined as the fractional number of years (over 1988–2010) for which at least one particle is located in each 0.5**°** (~50 km) grid square, and measuring the relative chance of encountering pumice rafts across the region at each time slice. Yellow star indicates Havre location.

From a hazard-forecasting perspective, we calculated the statistical likelihood of encountering pumice rafts in the vicinity of the Havre caldera volcano, using the 23 different patterns of simulated particle dispersal (for 1988–2010) within nominal ~50 km grid squares (Fig. 6). After 30 days, when the particles are little dispersed, particles drifting together in different directions and at different speeds for each year give rise to a small scatter of low likelihood. In contrast, particles are widely dispersed after 180 days and in some grid squares we find particles from up to 8 of the 23 years, equating to a likelihood of ~35%, considering that a likelihood of 100% corresponds to encountering particles from all 23 years in a given grid square. This indicates the areas where encounters with pumice rafts are most likely (for different elapsed times after the eruption) associated with the climatological mean surface currents and eddy stirring. Statistical assessment of risk could be refined to account for regional variations in the time-varying ‘concentration’ of particles (per unit area) in each year.

## Discussion

A number of important shipping routes pass near volcanic arcs (for example, Aleutians, Izu-Bonin, Mariana, Indonesia, Southwest Pacific and South Sandwich Islands). The spread of pumice rafts over thousands of km^2^ is common^12^, but is not currently forecast by any agency/country, and its late detection can result in economic losses to important shipping and fishing industries^11^,^12^,^15^,^22^. Future submarine eruptions and their products are more likely to be observed via satellite imagery than by humans^23^, and there will be delays between the eruption and its official assessment (for example, 3 weeks for Havre). There is a need for operational detection and forecasting of pumice raft dispersal to mitigate hazards by preparing communities and industries. This service does not currently exist, which is in contrast to the well-established Volcanic Ash Advisory Centres that provide detection and reporting to the International Civil Aviation Organization to mitigate risks from atmospheric ash plumes. As velocity fields are sampled from a global model, trajectory calculations can be repeated for any specific eruption, anywhere in the World Ocean, at short notice. Our high-fidelity simulations of particle drift and dispersal using eddy-resolving (1/12°) global ocean model hindcast demonstrate a novel method for future hazard analysis and warning for prediction of broad areas that may be affected by pumice rafts in the future, and can be used in conjunction with MODIS imagery on most parts of the oceans for not only rapid-response drift forecasts of pumice raft hazards but also for drift of anthropogenic waste, icebergs and marine organisms. For direct predictions, however, appropriate ocean forecast data and daily tracking of pumice rafts using satellite imagery are required. As an example, the UK National Centre for Ocean Forecasting uses NEMO for short-range (6-day) ocean forecasts. Global forecasts have been recently made available at the resolution necessary (1/12°) to simulate realistic dispersal of objects in the ocean^35^,^37^. Our model can thus be used to forecast the main raft components, which are the most disruptive.

Apart from direct hazards from the explosive eruption itself in the vicinity of an eruption column, which include entrance of pyroclastic flows into the sea, tsunamis, ballistics, fallout and eventual shoaling of a new volcanic island^18^,^20^,^48^,^49^,^50^,^51^, pumice rafts from such eruptions can block harbours^15^, and disrupt or divert coastal and transocean navigation/traffic over large areas at distances of thousands of kilometres from the eruption site over periods of months to years^9^,^15^,^20^,^21^,^22^. Water intake systems are critical components of any motorized vessel, and have been reported to get clogged by small pumice clasts during crossings of pumice rafts by both small and large watercraft^52^. For example, the yacht Maiken motored into a thin pumice raft in 2006 ‘…and within seconds Maiken slowed down from seven to one knot…we plowed a couple of hundred metres into this surreal floating stone field before we realized that we had to turn back. Just as we came out of the stone field and entered reasonably normal water we noticed that there was no cooling water coming from the engine…’^53^. Failure of a boat’s engine threatens all electric and electronic systems onboard, which are essential for navigation and communications. Hulls can also be damaged by abrasion. Environmental hazards from rafts include modification of the food web by killing photosynthetic organisms by shading, and by fostering and carrying invading exotic species^11^,^12^,^54^.

Marine ash beds are extremely useful stratigraphic markers^55^ since they can be dated and correlated over thousands of km^2^. In addition, thickness and dispersal of ash in oceans are often correlated with eruption style and magnitude, and used to infer distance and direction to the source vent^56^,^57^,^58^,^59^,^60^, as in on-land studies^61^. Estimations of eruption location and magnitude using marine ash-deposit dispersal can be problematic when thick voluminous pumice rafts are produced. The significance and origins of marine ash beds and pumice clasts on the sea floor have to be considered with caution. Pumice rafts will produce ash by a secondary process of comminution, whereby adjacent pumice clasts within the raft gently but repeatedly collide with one another. Attrition during rafting may generate widespread ash beds with facies that share some similarities with water-settled aerially transported ash. The extent of secondary ash beds from clast attrition in pumice rafts will depend on raft size, velocity and dispersal by oceanic surface currents rather than on eruption size and wind direction, and their distribution may not follow conventional isopach/isopleth ellipses of dispersal from atmospheric volcanic plumes. Even in cases where simple unidirectional dispersal takes place, isopach maps would reveal ocean current directions, and any calculation of eruption magnitude and atmospheric plume dynamics from such a distribution would be spurious. Raft-generated ash is not necessarily directly related to primary input from a subaerial or shallow water explosive eruption^25^. For the study of marine ash beds, complimentary analysis of the particles’ shapes, composition and of deposit sorting are critical, as these attributes can reveal a complex history of ash generation and transport processes, including rafting. Unlike ash previously sorted during subaerial floods off volcanic islands and/or transported in water-supported, seafloor-hugging density currents, raft-generated ash is likely to produce relatively poorly sorted marine ash beds, with crystals mixed with fine glass shards. Raft-generated ash will contain small pumice fragments and glass shards mixed with unbroken to slightly broken crystals (phenocrysts released from pumice clasts during low-energy comminution); a near-zero abundance of dense lithic grains in raft-generated ash contrasts strongly with water-settled fall deposits from ash delivered by atmospheric plumes. Ash settled from subaerial or submarine eruption plumes will contain glass shards, eruptively broken phenocrysts, and lithic clasts from conduit and vent walls. Importantly, raft-generated ash beds are likely to coexist with discrete outsized pumice clasts^62^, which sank from the rafts after reaching negative buoyancy in water through slow but continuous waterlogging.

**Figure 7:**
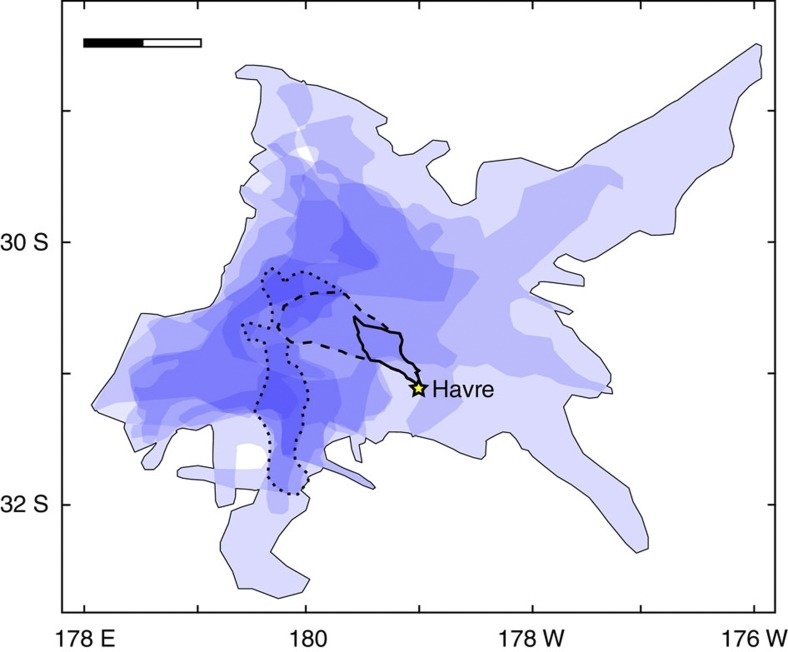
**Isopach map of raft-generated ash from the 2012 Havre eruption**. Data are compiled from stacked satellite images over the first 3 weeks of rafting (110,000 km^2^). Each shaded area is 3-day cumulated coverage of the rafts, taking raft movements between images into account. Shade intensity refers to amount of time where rafts were present in the area. Days 1–2 (thick black line, 1,290 km^2^), days 1–4 (dashed black line; 4,350 km^2^) and days 1–7 (dotted black line; 9,740 km^2^) show the expected area where most of the abrasion occurred. Effects of deep oceanic currents are neglected. Scale bar 100 km.

Until 6 August, MODIS images showed water discoloured (Fig. 2a,b) by the presence of ash and possible chemical compounds dissolved from ash^63^; pumice clasts collected in mid-August (3 weeks after eruption) were already rounded^31^. Comminution is most efficient on angular pyroclasts^64^, implying that secondary ash from the pumice rafts was chiefly produced during the first 3 weeks of drifting, and in particular the first week (Fig. 7). Over the first 3 weeks, the pumice rafts spread over a total area of 110,000 km^2^ (Supplementary Figs 1 and 2), and the estimated thickness of raft-generated ash beds expected to have accumulated on the sea floor averages 0.1–0.2 mm (see Methods), although the first days of rafting should have produced most of the ash^64^, generating much thicker beds, up to 15 mm thick, depending on the abrasion rate (see Methods) over many hundreds of km^2^. More accurate calculation of bed thickness is difficult because it is dependent on multiple unknown factors, such as abrasion rates (strongly dependent on wave motion) in this pumice raft, grain size distribution in the raft, raft thickness, and weather and wave heights. Unlike ash beds, seafloor deposits of pumice clasts that become sufficiently waterlogged and/or overloaded by sea life to sink^1^,^2^,^12^ consist of discrete clasts (with possible encrusting carbonate shells) spread over hundreds of thousands to millions of km^2^ on the sea floor, and are occasionally collected in piston cores^65^ and observed by submersible vehicles.

Millimetric to centimetric-sized pumice clasts from stranded pumice rafts are very rarely recognized in the geological record^2^,^3^,^66^,^67^,^68^,^69^,^70^, despite the rafts’ abundance over geological time (for example at least 17 marine pumice rafts in the last century^12^). If not washed onto high ground by tsunamis^68^,^70^ or large storms, pumice is quickly abraded in the tidal zone and rarely preserved. Stranded pumice raft deposits consist of distinctively rounded pumice clasts, although coarse clasts may float on a preferred (more stable) side, their top part remaining subangular^67^; lithic clasts are absent from the deposit. Long-drifting clasts will be colonized by marine life and encrusted by shells^12^, particularly in tropical latitudes. If stranded on high ground, pumice clasts may be reworked by surficial water, resulting in further modification of their shape and of the depositional facies. As comparison, lacustrine pumice raft deposits consist of well-rounded pumice clasts interbedded with relatively thick beds of ash^2^,^28^,^29^, reflecting extensive attrition of pumice clasts during rafting due to the confined environment preventing their gradual dispersion, in addition to shoreline-derived abrasion; giant blocks of pumice are present locally.

## Methods

### Hindcast model

The NEMO model^43^ was used for hindcasting. NEMO was used in eddy-resolving (1/12°) configuration to provide 5-day averages of the eddying surface currents, a time window appropriate to model realistically and with high precision the advection of an ensemble of particles mimicking pumice clasts released from this volcano. We simulate release of particles above the Havre location on 18 July in each year of the period 1988–2010, and then produce a composite trajectory ensemble representing average current motions in the Havre region. To mimic pumice behaviour, modelled particles are specified as buoyant and the simulated trajectories are thus subject only to surface advection. We consider that pumice clasts that sank by waterlogging did not affect the raft’s dispersal pattern. The sampled current field evolves annually in response to seasonal and interannual variability of geostrophic and Ekman currents, and is also subject to chaotic eddying. We neglect normal forces and tangential stresses due to the wind acting on the pumice raft itself, as well as any changes in winds and currents due to anomalous vertical momentum fluxes associated with the presence of the rafts themselves.

### Raft volume

The maximum raft thickness from isolated point measurements^31^ was 60 cm on 10 August. Taking this thickness into account, and from colour radiance and brightness on MODIS images over the same day, we estimate that the thickest part of the raft (averaged at 50 cm) was spread over 200 km^2^, and that this main thick raft was surrounded by a 10–20-cm-thick raft edge covering 1,000 km^2^. From this, the total pumice raft volume is estimated at 0.18–0.26 km^3^. Considering that the isolated point measurements^31^ are unlikely to have been taken in the thickest part of the raft, and that many pumice clasts may have sunk^1^,^2^,^30^ or were partly abraded in the first weeks of drifting following their partial waterlogging during the eruption, a total pumice raft volume up to five times larger (that is, 1 km^3^) is more realistic than these minimum values. The 1-day-old post-eruption minimum raft thickness of 50–70 cm after the eruption is calculated by dividing the minimum total pumice raft volume (0.18–0.26 km^3^) per the raft area on the 19 July UTC (400 km^2^). Considering a realistic 1 km^3^ volume (see above), the 1-day-old post-eruption expected raft thickness would approximate 2.5–3.5 m, which is not exceptional^18^.

A packing of clasts at 60% of solids gives a minimum total rafted pumice volume of 0.11–0.16 or 0.03–0.05 km^3^ dense rock equivalent. From image analysis on SEM images of pumice clasts, an average vesicularity of 70% is used for dense rock equivalent calculations. Density of clasts per m^3^ follows previous methods^12^.

### Thickness of raft-generated ash beds

Discoloured water, which is a sign of sediment (ash) in water^63^, was seen surrounding the raft until 6 August on MODIS images. The mid-August (3 weeks post eruption) pumice raft samples were reported as rounded^31^. A pumice clast becomes rounded by losing 20–40% of its volume^64^ to abrasion. The average thickness of a seafloor ash bed (0.1–0.2 mm) produced only by comminution is estimated by dividing the volume of comminuted pumice (20–40 vol% of the total rafted pumice volume, see above) by the cumulated dispersal area (110,000 km^2^) of the raft until the morning of 9 August (UTC). Comminution of pumice mostly produces non-to-poorly vesicular glass shards, and their packing in ash beds is estimated to be 60% solids. Favouring quick pumice abrasion^64^, raft-generated ash beds are likely to be 1–2 mm thick if most of the abrasion occurred during the first week of drifting (cumulated spread of 9,740 km^2^), or 5–15 mm thick in the case abrasion chiefly occurred in the first 2 days (cumulated spread of 1,290 km^2^). The dispersal and reworking of ash by deep and bottom water currents is neglected in these estimations.

## Additional information

**How to cite this article:** Jutzeler, M. *et al.* On the fate of pumice rafts formed during the 2012 Havre submarine eruption. *Nat. Commun.* 5:3660 doi: 10.1038/ncomms4660 (2014).

## Supplementary Material

Supplementary Information Supplementary Figures 1-30
