# Supplementary material for: On the fate of pumice rafts formed during the 2012 Havre submarine eruption
Source: Nat Commun. 2014 Apr 22;5:3660. doi: 10.1038/ncomms4660 (PMC3997806; doi:10.1038/ncomms4660)
Supplement: Supplementary Information — Supplementary Figures 1-30 [file ncomms4660-s1.pdf]

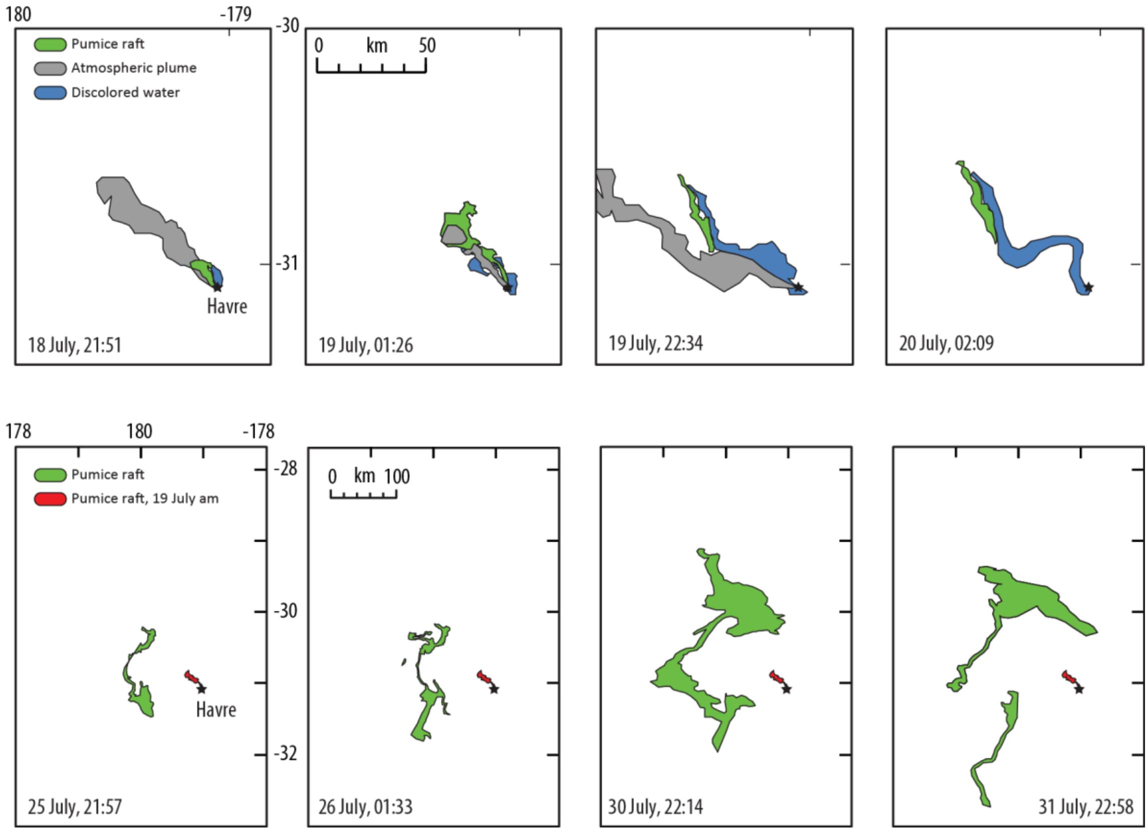

Supplementary Fig. 1 Detailed sequence of the Havre pumice raft, from analysis of MODIS images for July 2012. All times in UTC. Cloud cover may reduce raft and plumes visbility.

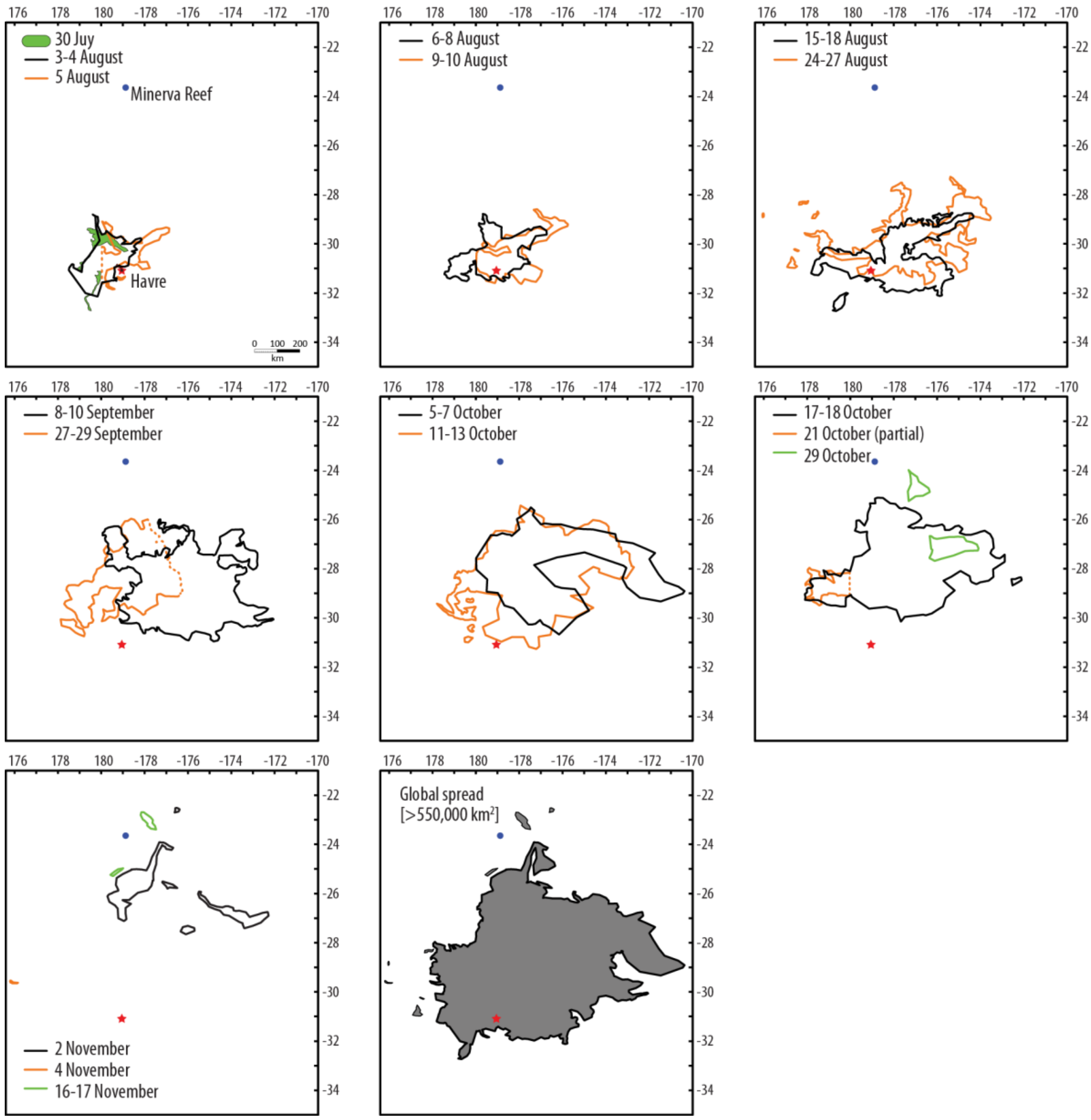

Supplementary Fig. 2 Sequence of the Havre 2012 pumice raft, from analysis of MODIS images for August-November 2012. Polygons represent global area where rafts are present. All times in UTC. Cloud cover may reduce raft visbility.

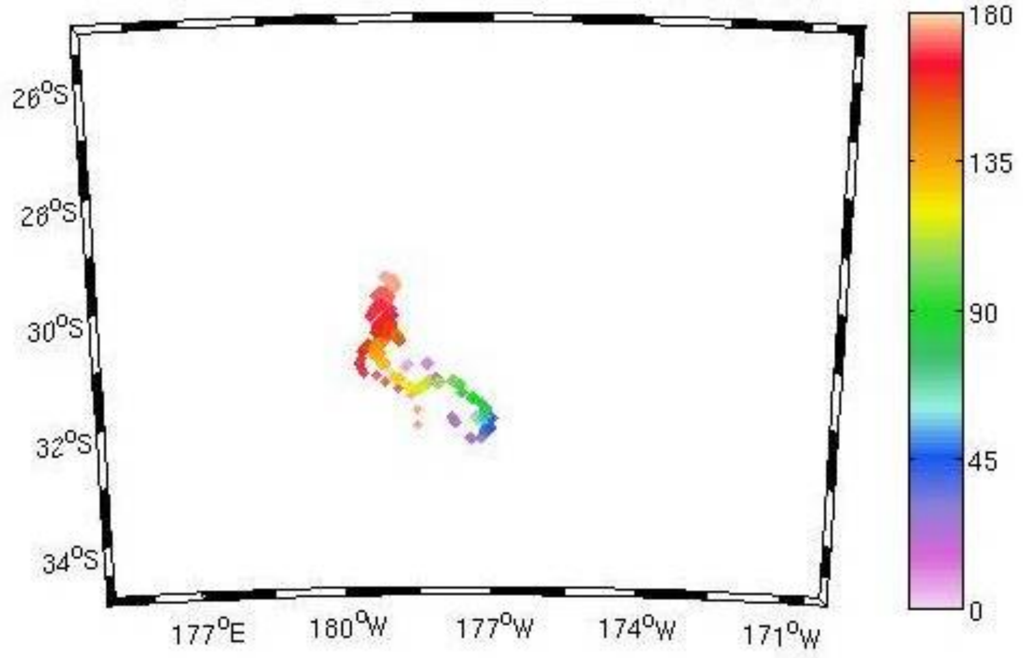

Supplementary Fig. 3 2400 particles starting at 18/7/1988, colour-coded by age (days)

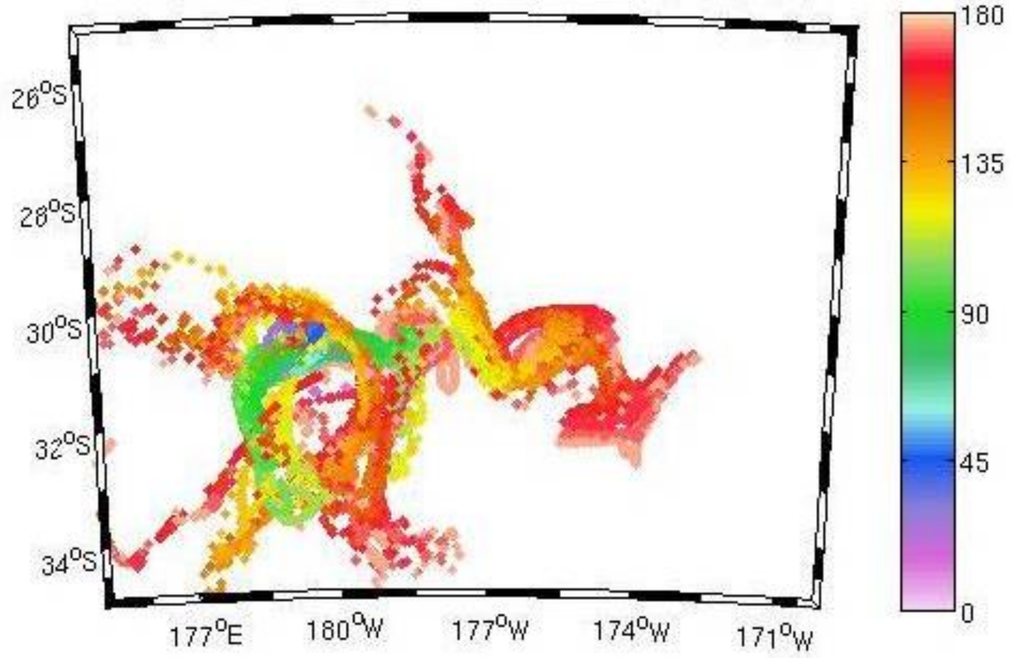

Supplementary Fig. 4 2400 particles starting at 18/7/1989, colour-coded by age (days)

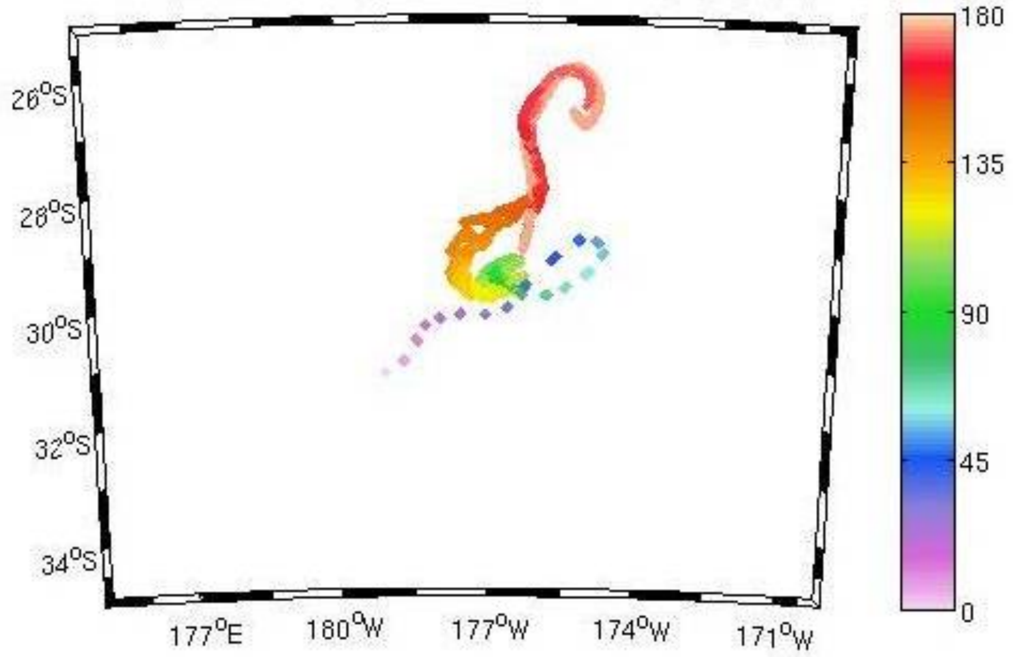

Supplementary Fig. 5 2400 particles starting at 18/7/1990, colour-coded by age (days)

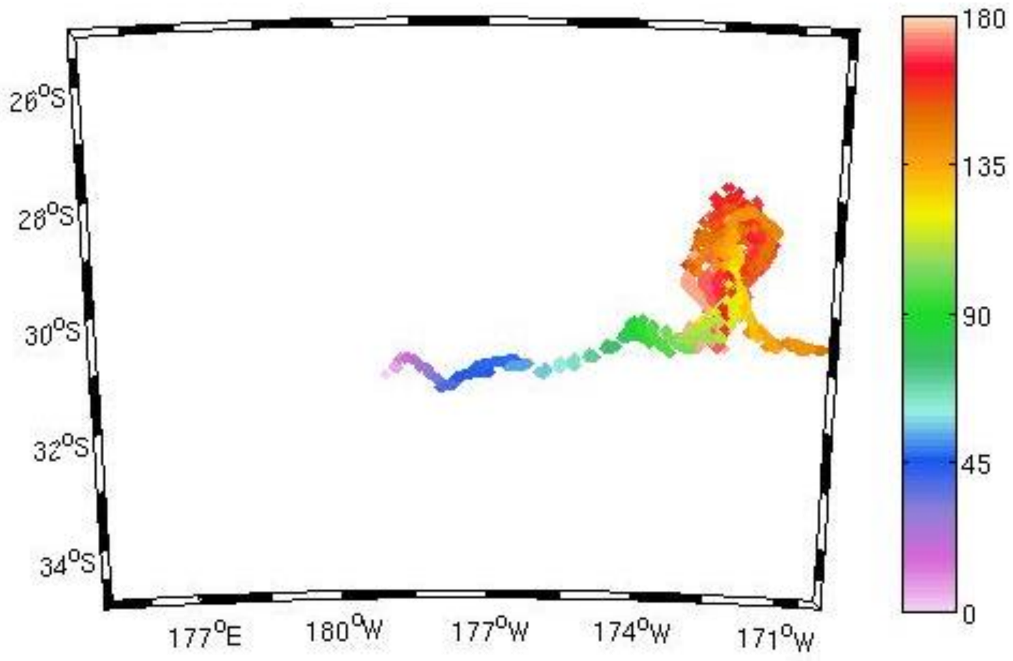

Supplementary Fig. 6 2400 particles starting at 18/7/1991, colour-coded by age (days)

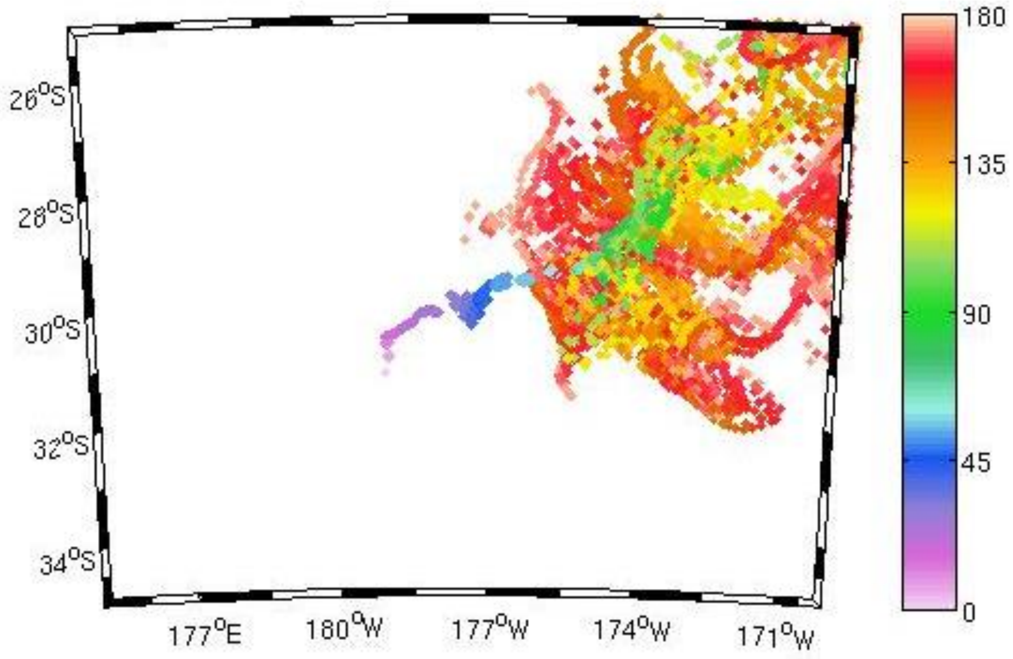

Supplementary Fig. 7 2400 particles starting at 18/7/1992, colour-coded by age (days)

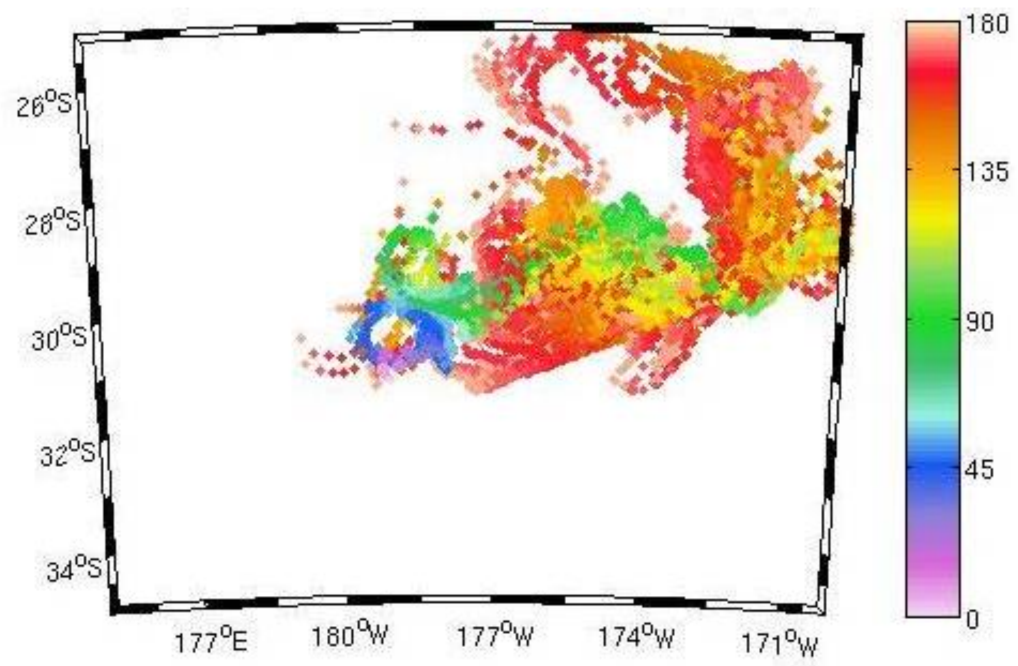

Supplementary Fig. 8 2400 particles starting at 18/7/1993, colour-coded by age (days)

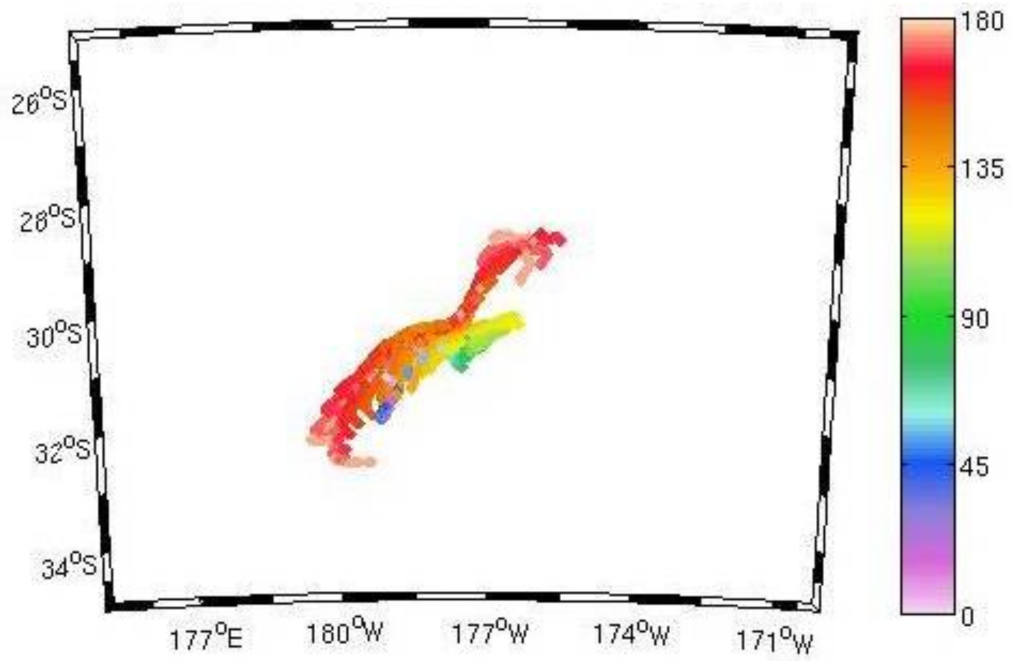

Supplementary Fig. 9 2400 particles starting at 18/7/1994, colour-coded by age (days)

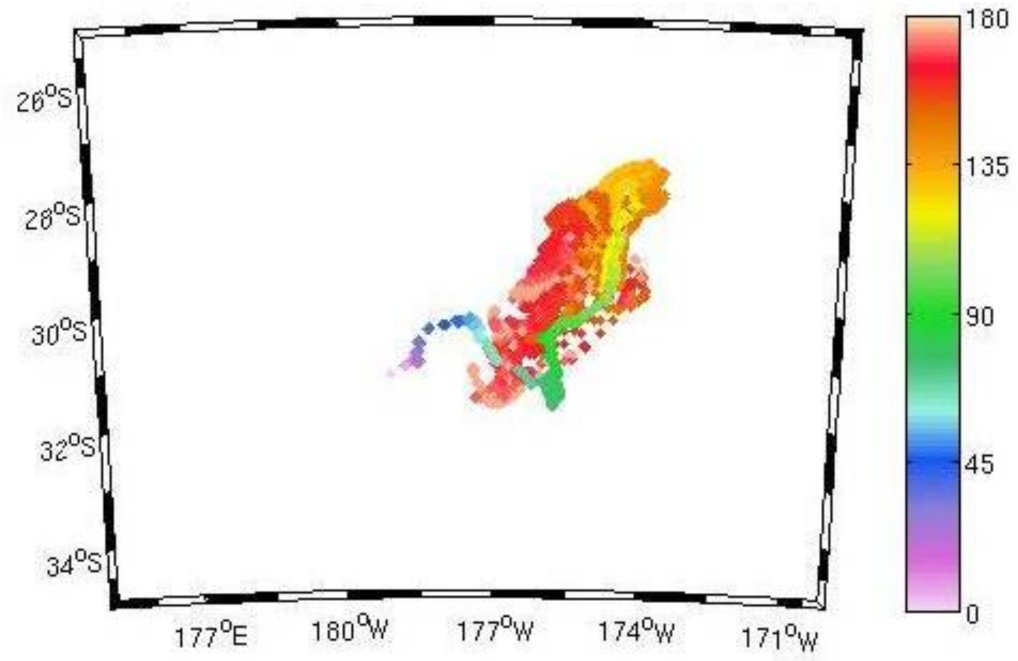

Supplementary Fig. 10 2400 particles starting at 18/7/1995, colour-coded by age (days)

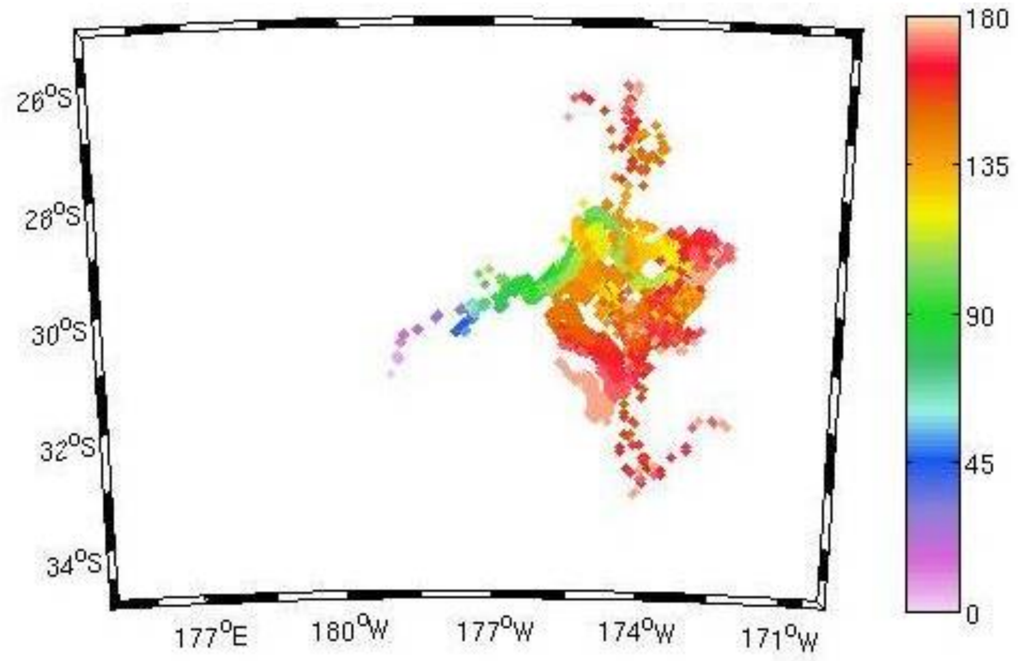

Supplementary Fig. 11 2400 particles starting at 18/7/1996, colour-coded by age (days)

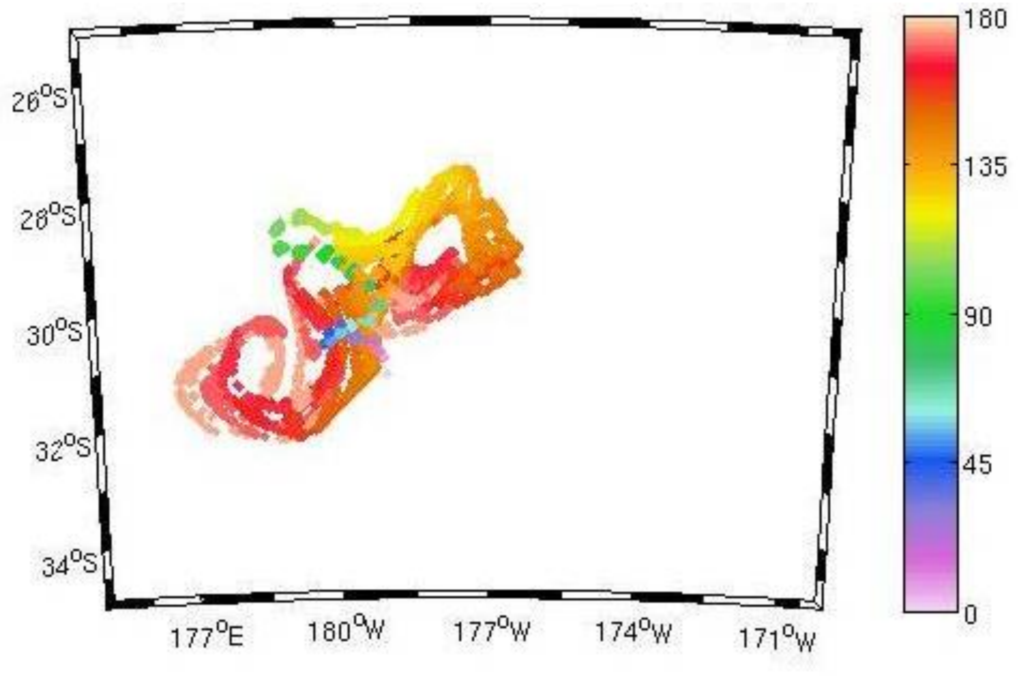

Supplementary Fig. 12 2400 particles starting at 18/7/1997, colour-coded by age (days)

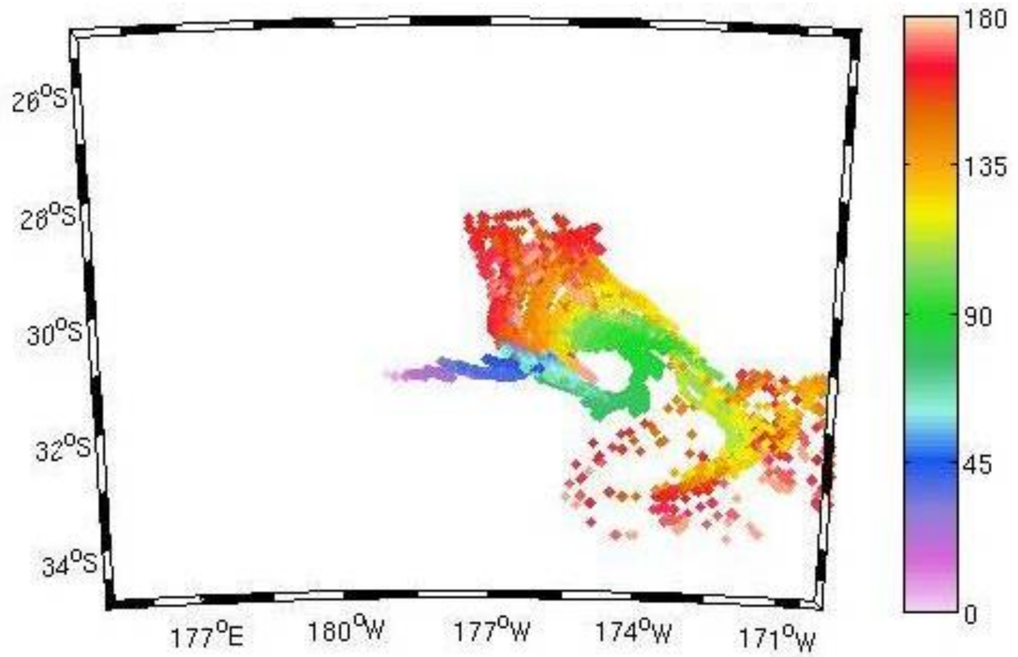

Supplementary Fig. 13 2400 particles starting at 18/7/1998, colour-coded by age (days)

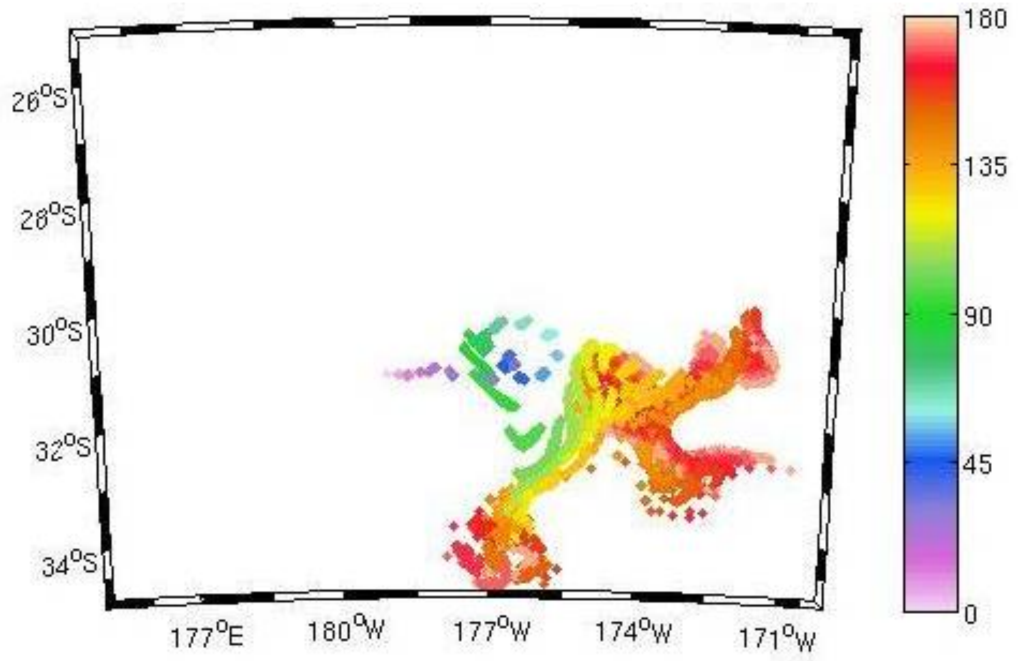

Supplementary Fig. 14 2400 particles starting at 18/7/1999, colour-coded by age (days)

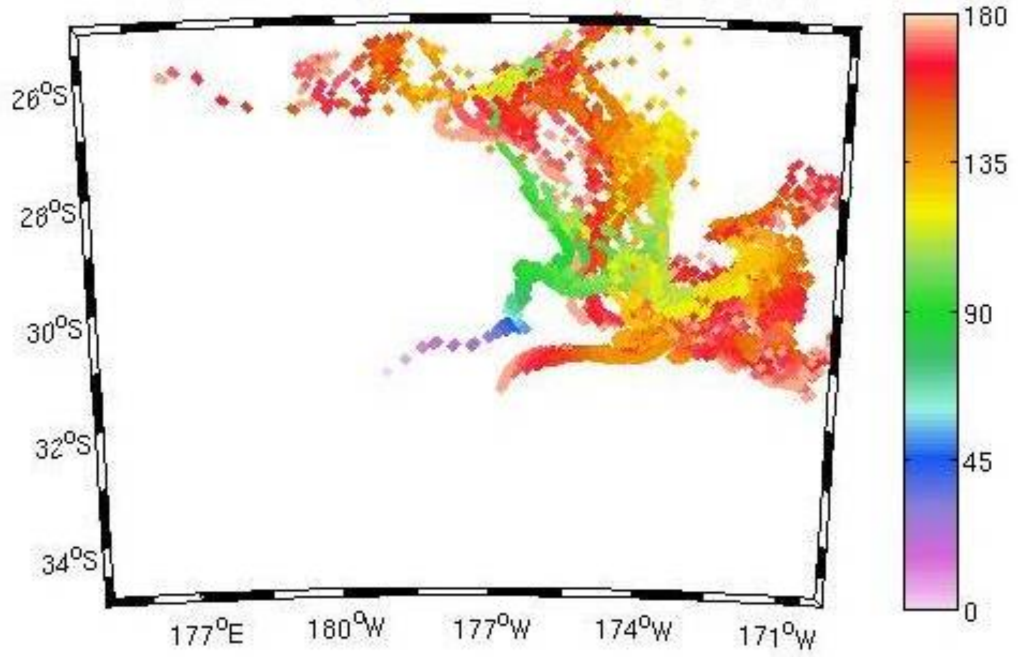

Supplementary Fig. 15 2400 particles starting at 18/7/2000, colour-coded by age (days)

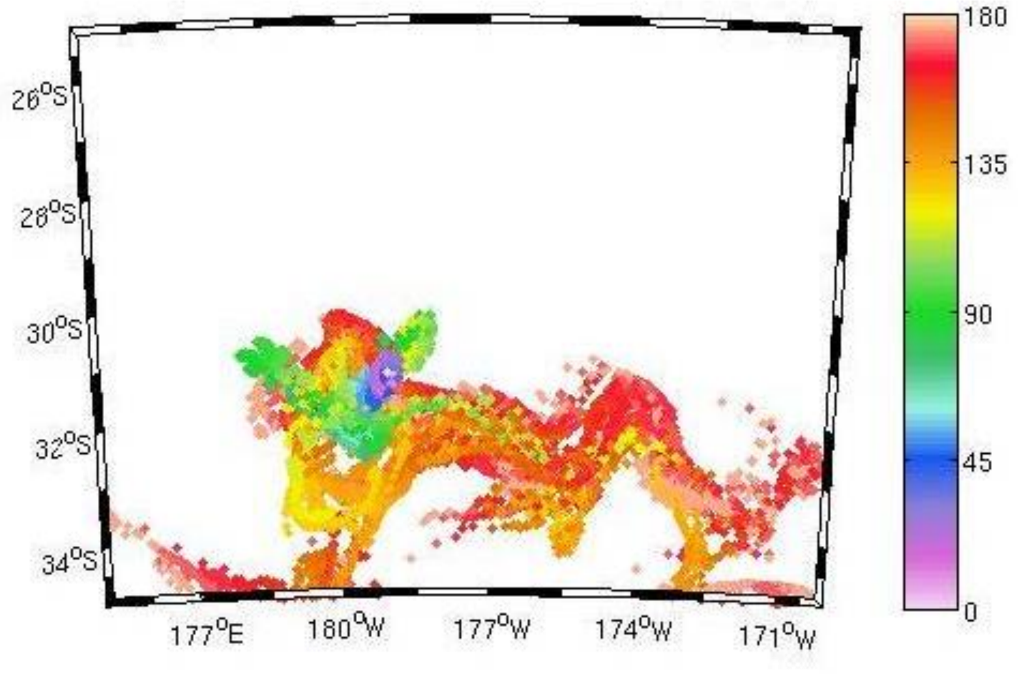

Supplementary Fig. 16 2400 particles starting at 18/7/2001, colour-coded by age (days)

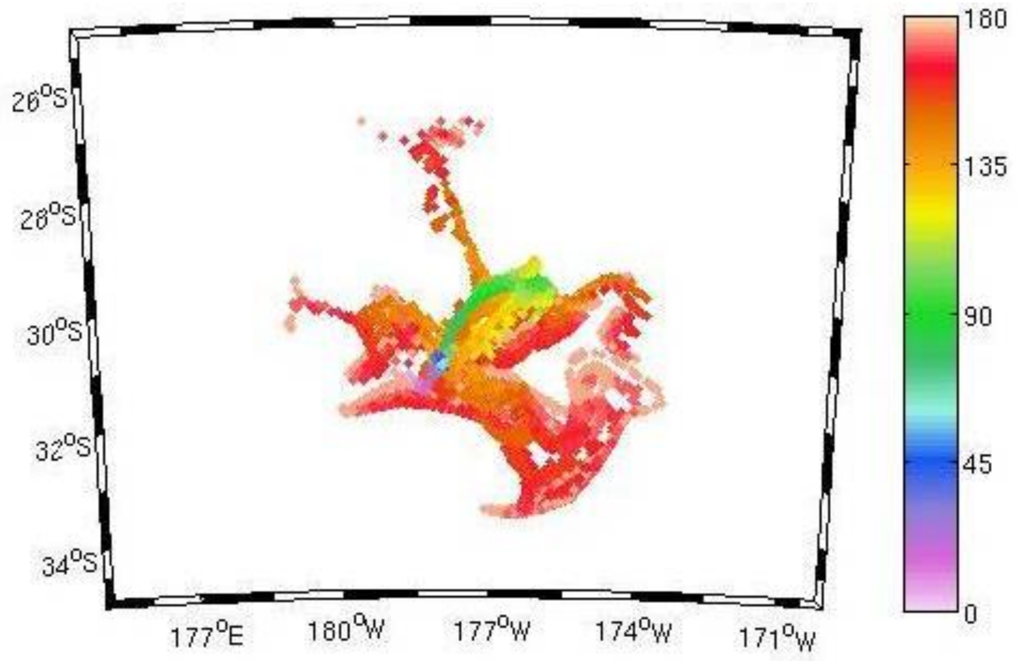

Supplementary Fig. 17 2400 particles starting at 18/7/2002, colour-coded by age (days)

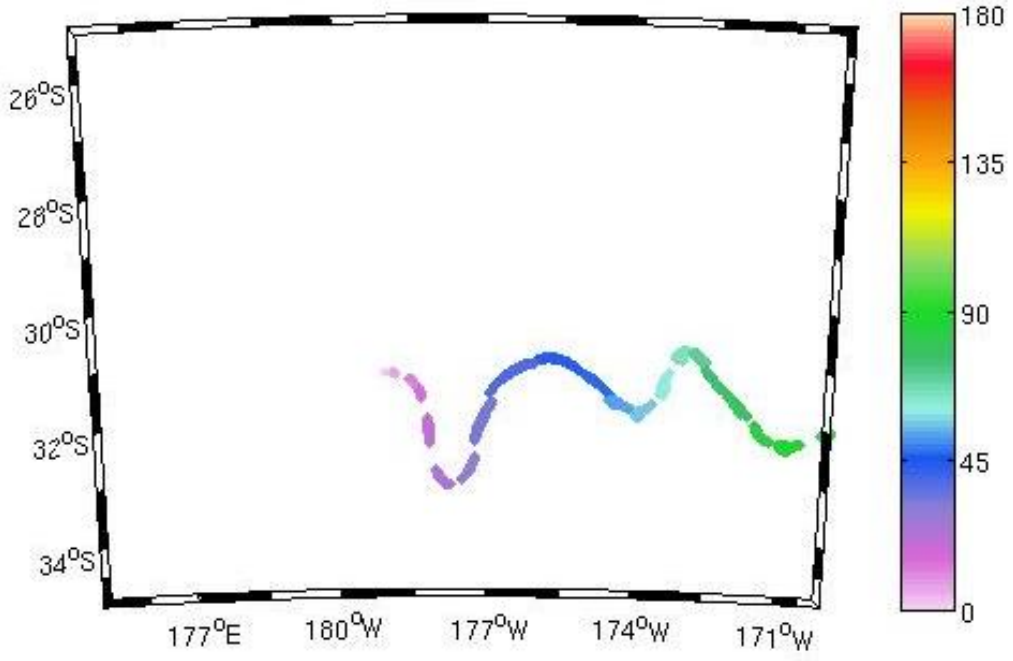

Supplementary Fig. 18 2400 particles starting at 18/7/2003, colour-coded by age (days)

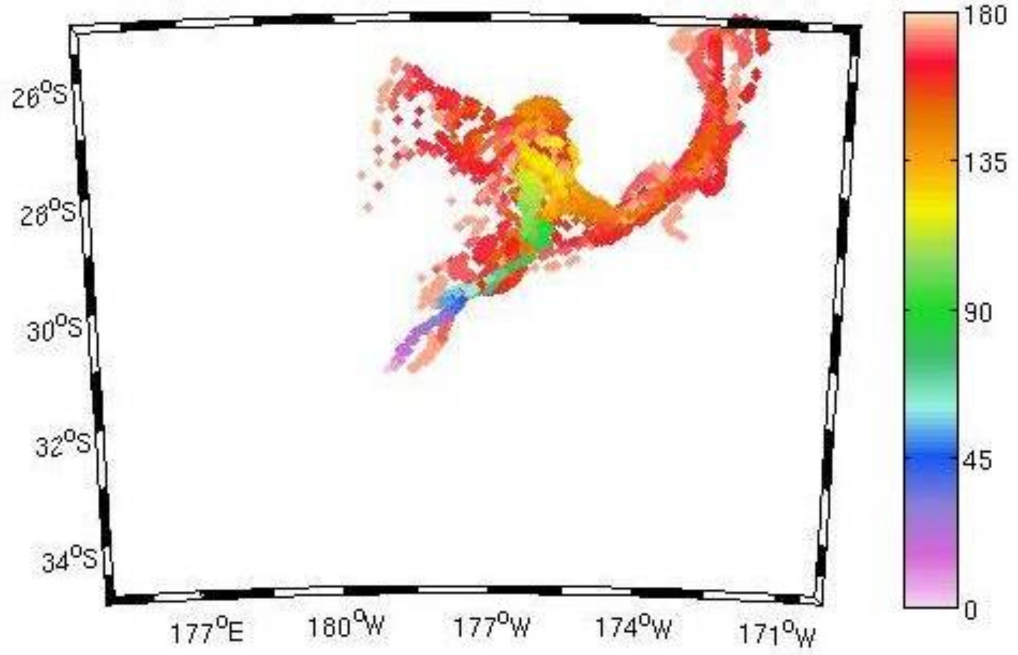

Supplementary Fig. 19 2400 particles starting at 18/7/2004, colour-coded by age (days)

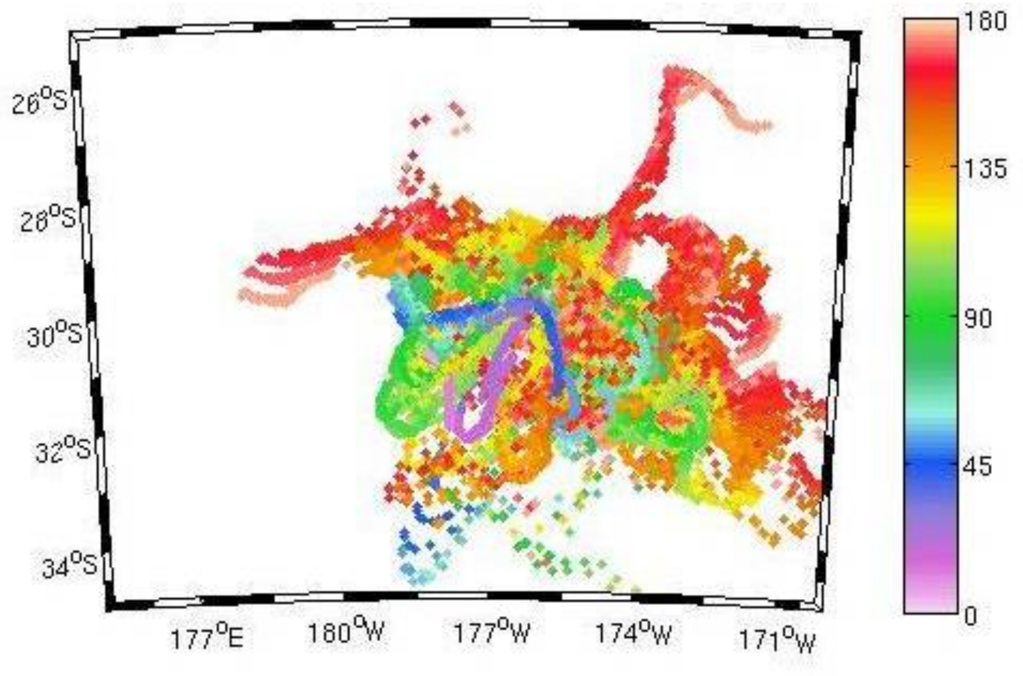

Supplementary Fig. 20 2400 particles starting at 18/7/2005, colour-coded by age (days)

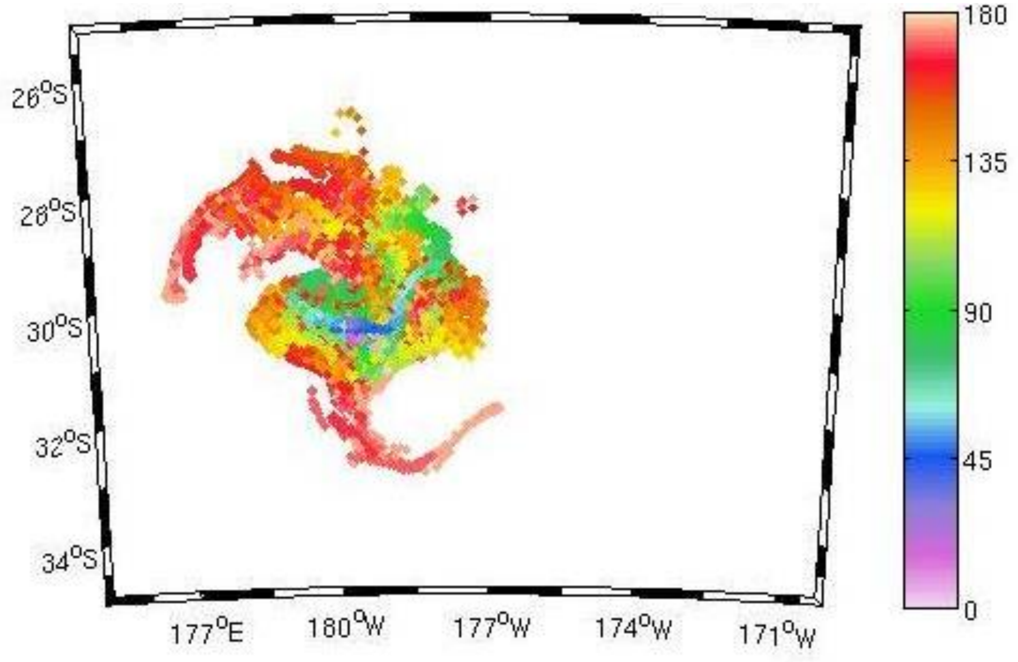

Supplementary Fig. 21 2400 particles starting at 18/7/2006, colour-coded by age (days)

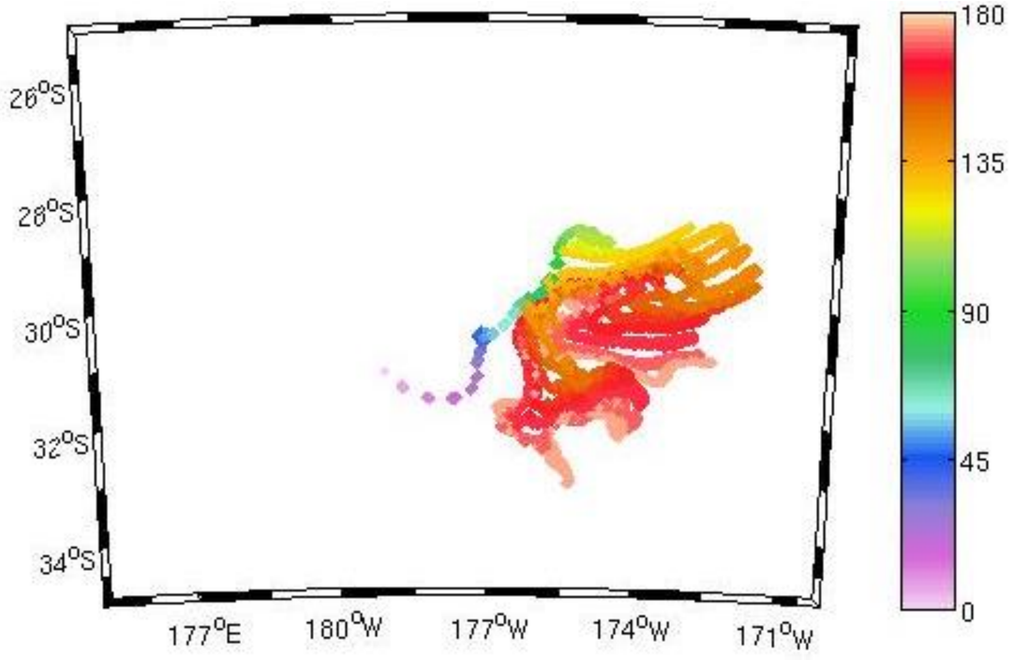

Supplementary Fig. 22 2400 particles starting at 18/7/2007, colour-coded by age (days)

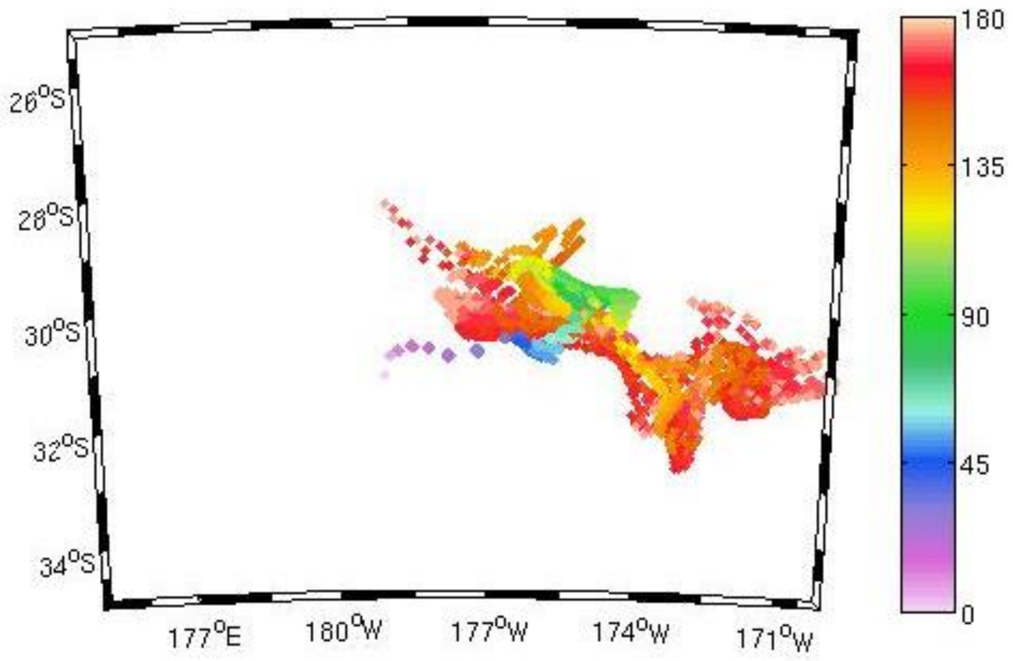

Supplementary Fig. 23 2400 particles starting at 18/7/2008, colour-coded by age (days)

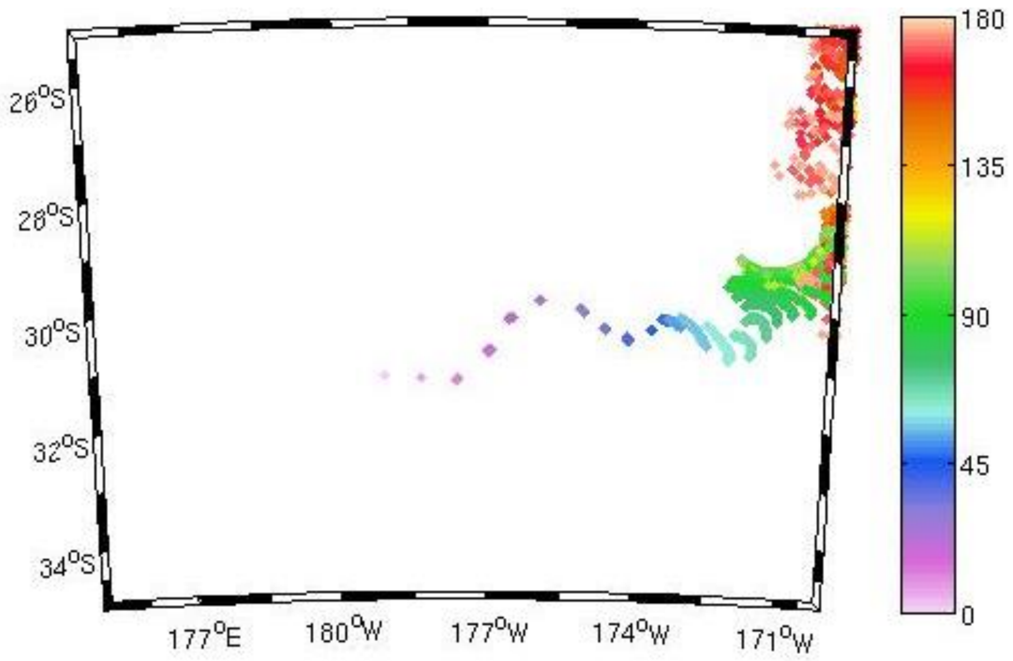

Supplementary Fig. 24 2400 particles starting at 18/7/2009, colour-coded by age (days)

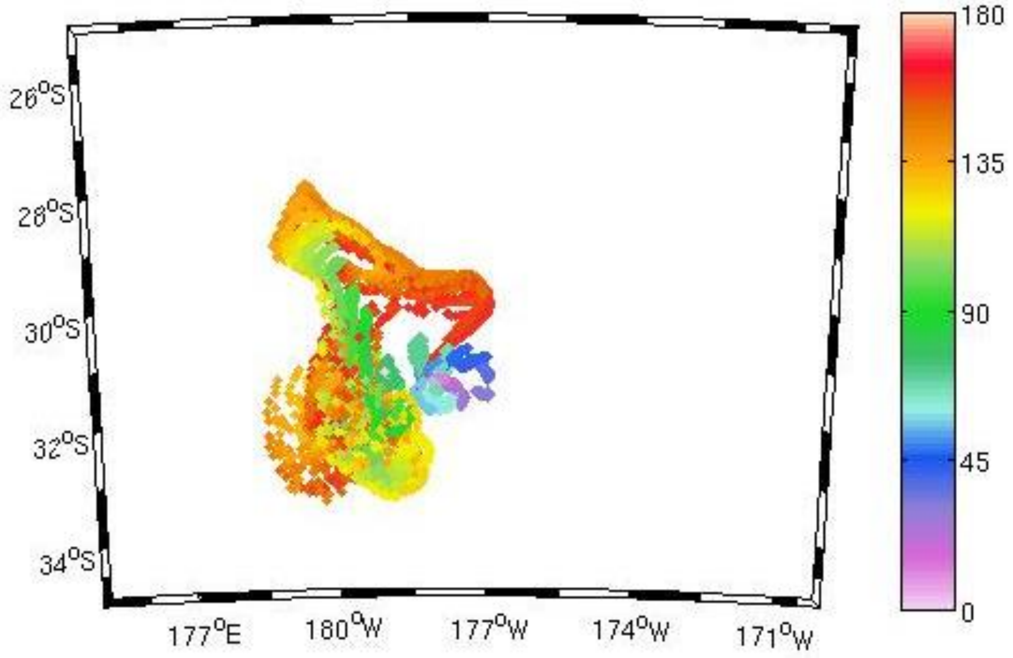

Supplementary Fig. 25 2400 particles starting at 18/7/2010, colour-coded by age (days)

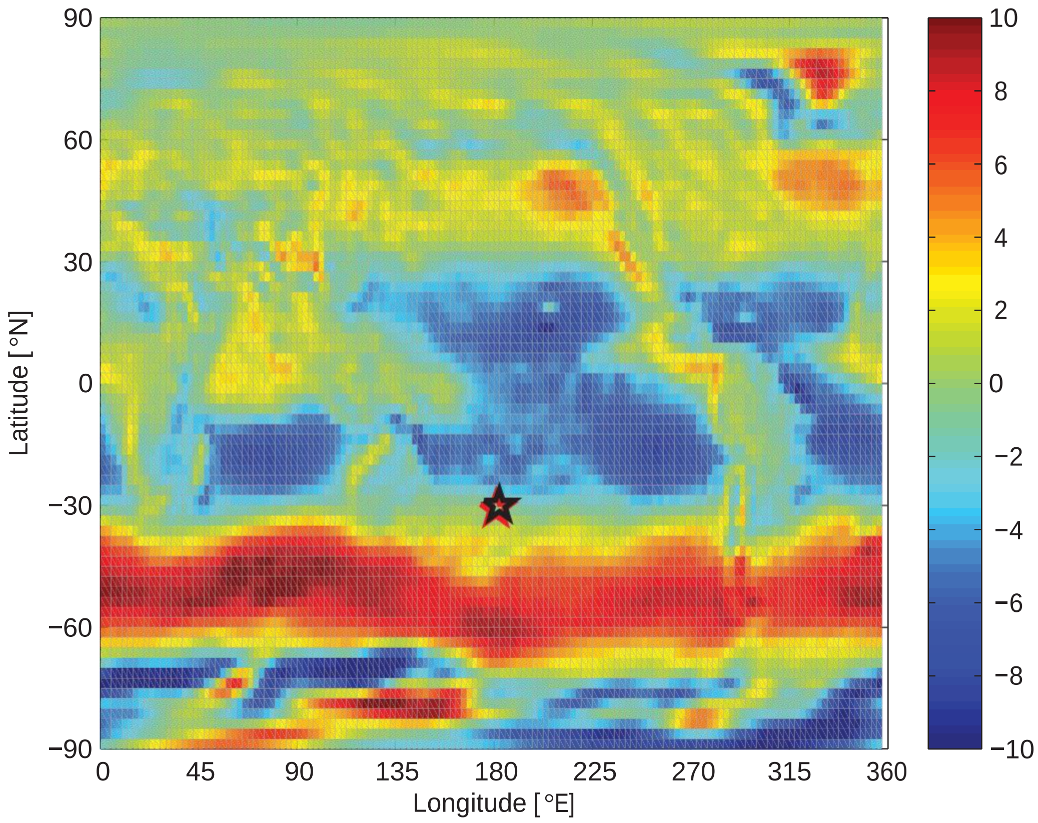

Supplementary Fig. 26 NCEP/NCAR re−analysis project. E−W wind component (m/s) – 18/07/12–31/12/12 mean

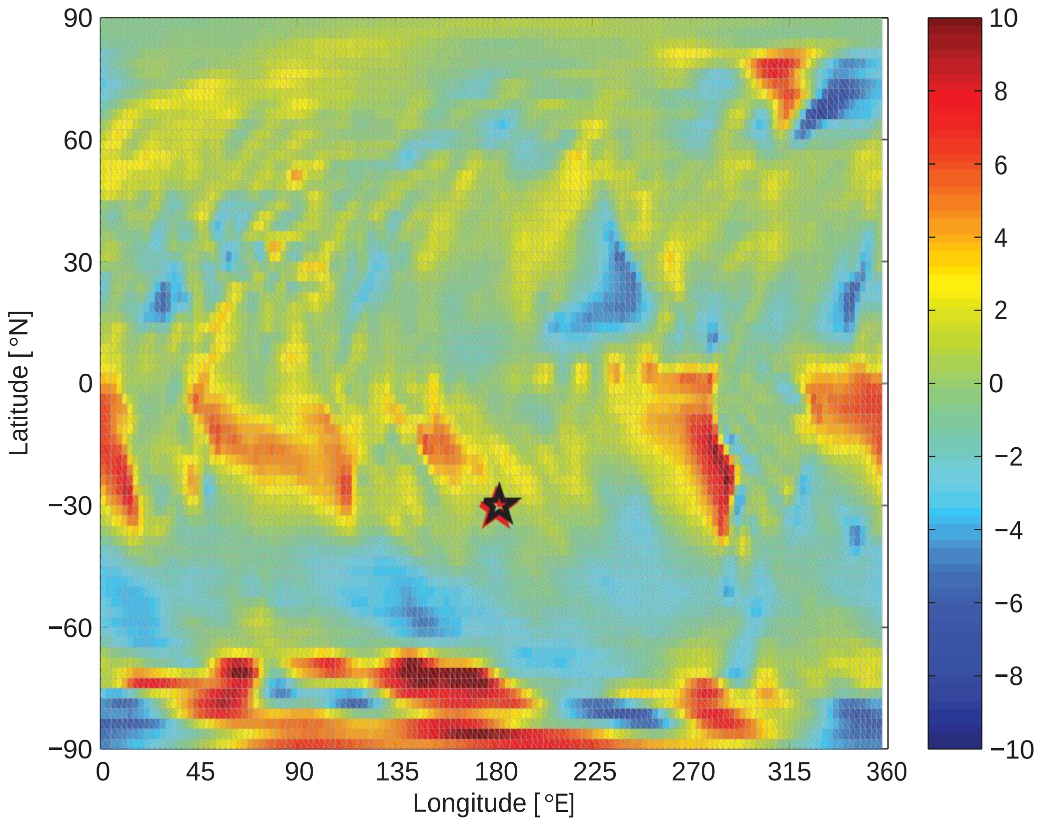

Supplementary Fig. 27 NCEP/NCAR re−analysis project. N−S wind component (m/s) – 18/07/12–31/12/12 mean

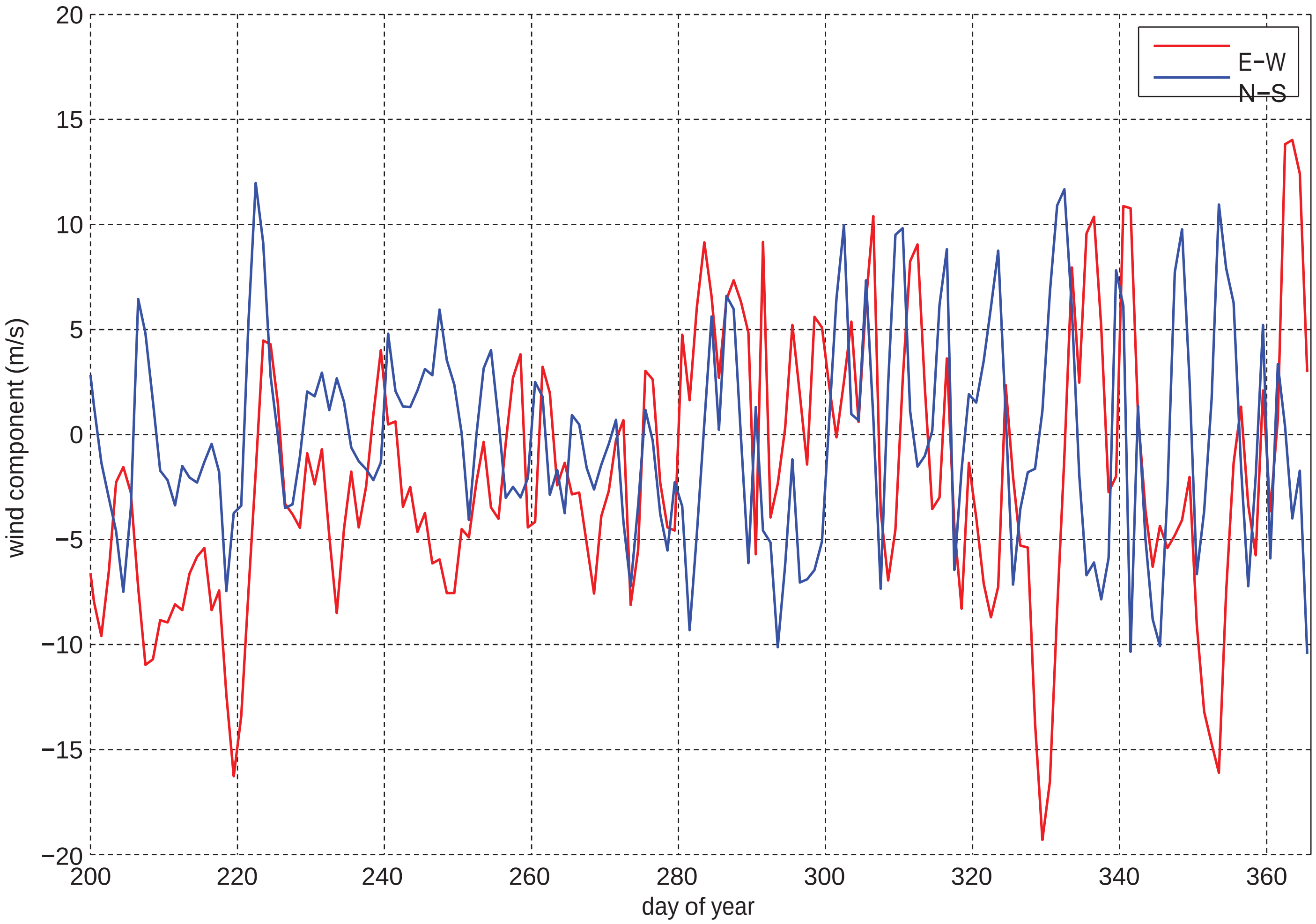

Supplementary Fig. 28 Daily−averaged wind components at 2.5 deg square centred 30S, 177.5W; 18/07/12−31/12/12

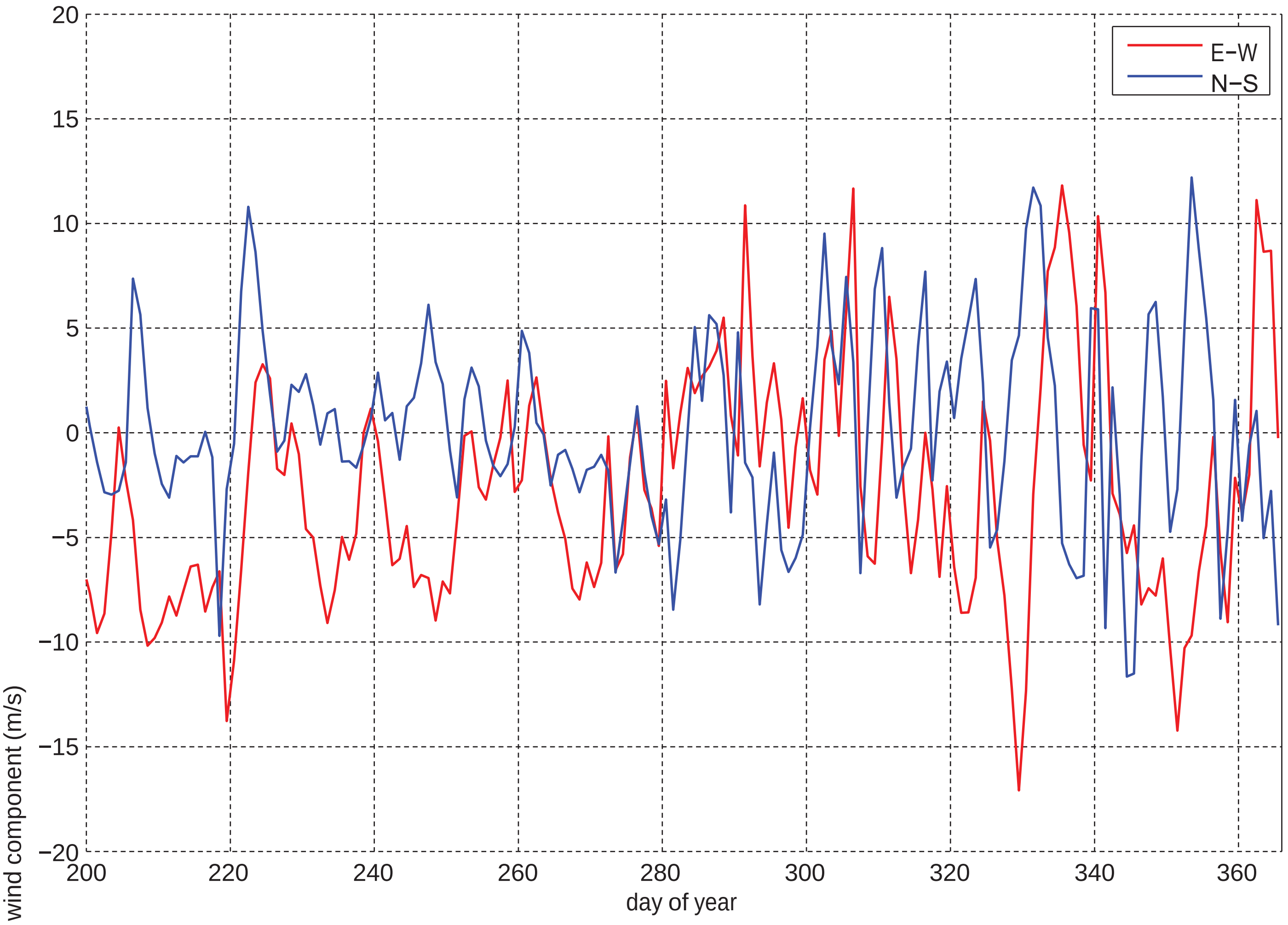

Supplementary Fig. 29 Daily−averaged wind components at 2.5 deg square centred 27.5S, 177.5W; 18/07/12−31/12/12

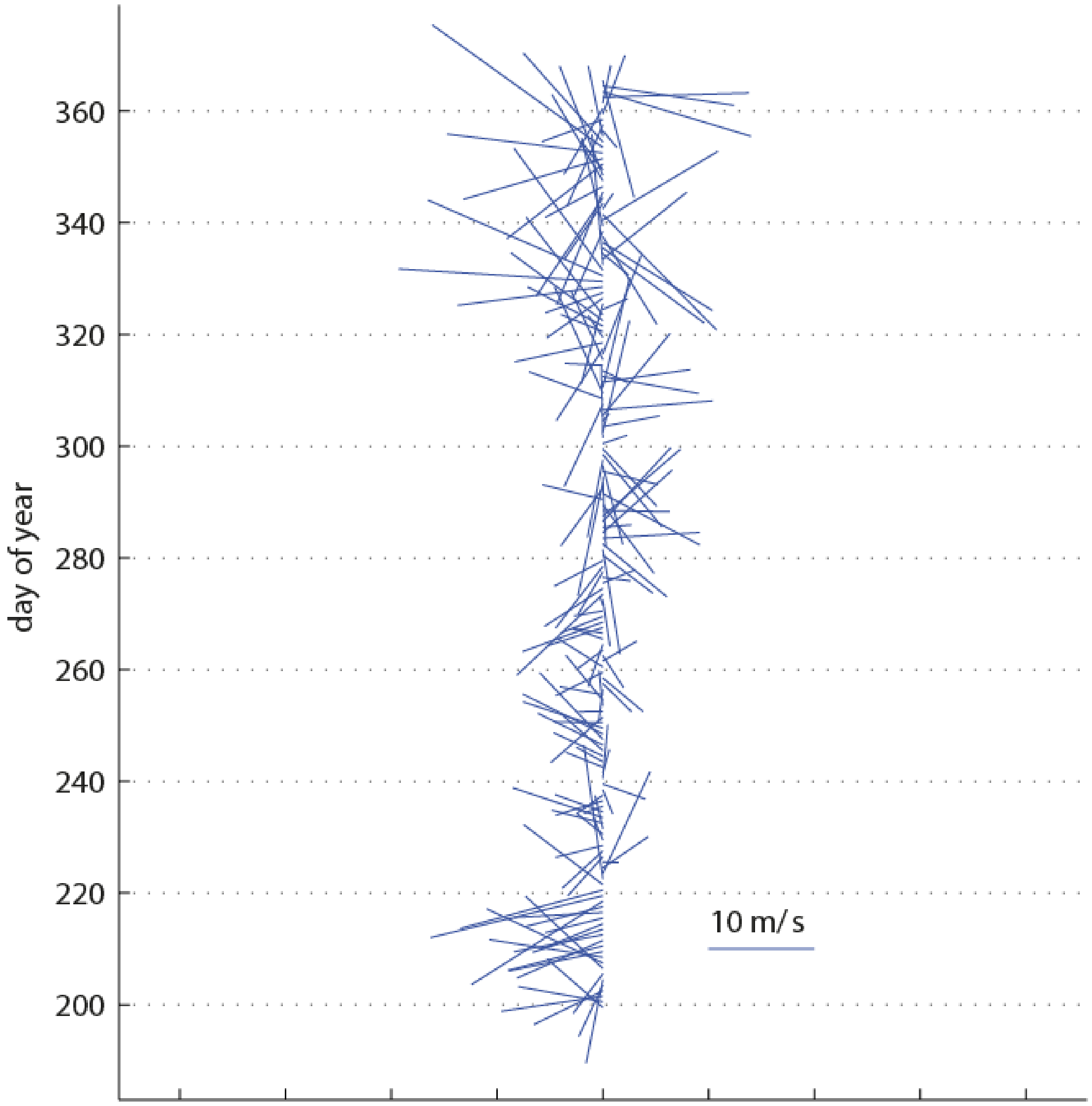

Supplementary Fig. 30 Daily–averaged wind vector at 2.5 deg square centred 30S, 177.5W; 18/07/12–31/12/12
